# *In silico*, *in vitro* and *in vivo* safety evaluation of *Limosilactobacillus reuteri* strains ATCC PTA-126787 & ATCC PTA-126788 for potential probiotic applications

**DOI:** 10.1371/journal.pone.0262663

**Published:** 2022-01-26

**Authors:** Dharanesh Gangaiah, Shrinivasrao P. Mane, Nilesh R. Tawari, Nallakannu Lakshmanan, Valerie Ryan, Alyssa Volland, Dwi Susanti, Milind Patel, Abraham Abouzeid, Emily B. Helmes, Arvind Kumar

**Affiliations:** Elanco Animal Health, Greenfield, Indiana, United States of America; Technical Educational Institute of Peloponnese, GREECE

## Abstract

The last two decades have witnessed a tremendous growth in probiotics and in the numbers of publications on their potential health benefits. Owing to their distinguishing beneficial effects and long history of safe use, species belonging to the *Lactobacillus* genus are among the most widely used probiotic species in human food and dietary supplements and are finding increased use in animal feed. Here, we isolated, identified, and evaluated the safety of two novel *Limosilactobacillus reuteri (L*. *reuteri*) isolates, ATCC PTA-126787 & ATCC PTA-126788. More specifically, we sequenced the genomes of these two *L*. *reuteri* strains using the PacBio sequencing platform. Using a combination of biochemical and genetic methods, we identified the two strains as belonging to *L*. *reuteri* species. Detailed *in silico* analyses showed that the two strains do not encode for any known genetic sequences of concern for human or animal health. *In vitro* assays confirmed that the strains are susceptible to clinically relevant antibiotics and do not produce potentially harmful by-products such as biogenic amines. *In vitro* bile and acid tolerance studies demonstrated that the two strains have similar survival profiles as the commercial *L*. *reuteri* probiotic strain DSM 17938. Most importantly, daily administration of the two probiotic strains to broiler chickens in drinking water for 26 days did not induce any adverse effect, clinical disease, or histopathological lesions, supporting the safety of the strains in an *in vivo* avian model. All together, these data provide *in silico*, *in vitro* and *in vivo* evidence of the safety of the two novel candidates for potential probiotic applications in humans as well as animals.

## Introduction

The term “probiotic” was derived from “pro” (Latin, means “for”) and “bios” (Greek, means “life”) and thereby means “for life”. Probiotics are defined as “live microorganisms that, when administered in adequate amounts, confer a benefit on the host” [1]. In recent years, there has been an unprecedented growth in the application of probiotics to support health and well-being. Often consumed as dietary supplements, nutraceuticals or as part of functional foods, probiotics are associated with many health benefits in the form of promoting gut barrier function, including studies on their potential to prevent and/or treat gastrointestinal diseases, inhibit pathogenic bacteria, and favourably modulate gut bacteria, the immune system, and host metabolism [2–5]. Several species of microorganisms are used as probiotics and the lactic acid bacteria belonging to the *Lactobacillus* genus, first described in 1901 [6], are among the most commonly used and well-studied probiotic bacteria with a long history of safe use [7].

*Limosilactobacillus reuteri (L*. *reuteri)*, a member of the *Lactobacillus* genus, are Gram positive, non-spore forming, non-motile bacteria, which are naturally adapted to survive under low pH, bile-rich, and microaerophilic to strictly anaerobic gastrointestinal environments [8]. German microbiologist Gerhard Reuter first isolated *L*. *reuteri* from human fecal and intestinal samples and classified it as *L*. *fermentum* biotype II [9]; later, Kandler *et al*., (1980) identified *L*. *reuteri* as a distinct species [10]. *L*. *reuteri* is considered one of the few true autochthonous lactobacilli present frequently in the gastrointestinal tract of all vertebrates, including humans, monkeys, chicken, turkeys, doves, pigs, dogs, lambs, cattle and rodents [11, 12]. *L*. *reuteri* strains are often known to produce reuterin (a bacteriocin with antimicrobial properties), cobalamin and folate, exclude or inhibit pathogens, modulate immune response, and enhance gut barrier function [13–17]. Several clinical studies have been published on the efficacy of *L*. *reuteri* in treating gastrointestinal disorders such as infantile colic, regurgitation, functional constipation, abdominal pain, and necrotizing enterocolitis [17–22]. *L*. *reuteri* was used in sourdough bread in 1980, and was introduced into human functional foods as a starter in the production of a special drink called “BRA (stands for *B**ifidobacterium*, *R**euteri* and *A**cidophilus*)” and a fermented milk called “BRA fil” in 1991 in Sweden [23]. Since then, *L*. *reuteri* strains, such as DSM 17938 and RC-14, have been widely used as a part of many commercially available dietary supplements and functional foods [17].

Lactic acid bacteria are known for their safety and are one of the probiotic microbial types with the longest history of safe use [7]. The *L*. *reuteri* species is usually considered safe for human and animal consumption due to the facts that they have been used as part of fermented foods for more than 30 years, they are normal inhabitants of the human and animal gut microflora, and they have regulatory stature in the United States and the European Union. Indeed, several strains belonging to the *L*. *reuteri* species were notified to the United States Food and Drug Administration (FDA), including three strains as “Generally Regarded As Safe (GRAS)” for use in specific foods and two strains as new dietary ingredients; the FDA cited no objections to such strains [24–28]. Moreover the *L*. *reuteri* strains was granted “Qualified Presumption of Safety (QPS)” status and is considered safe for use in, or as a source of food for, human and animal consumption by European Food Safety Agency (EFSA) [24–26, 29, 30]. A growing number of clinical studies have repeatedly confirmed the safety of *L*. *reuteri* not only in healthy individuals but also in immunocompromised individuals such as those positive for HIV [31, 32].

Despite the prior safe use of a probiotic genus and species, the survival properties, efficacy, and safety of probiotics are evaluated on a strain-specific basis. Hence, screening for such properties for every new strain is required before any new probiotic candidate is accepted for human and animal consumption. Hence, various regulatory agencies and experts have established comprehensive recommended guidelines for efficacy and safety assessment of new probiotic candidates [33–35]. These guidelines encompass a series of *in vitro*, *in silico* and *in vivo* studies, of which genomics is considered a powerful tool for rapid screening of probiotic candidates for safety.

Genomic characterizations are instrumental in selecting a safe and efficacious probiotic strain. Safety assessment begins with the correct identification of the probiotic candidate and this is important for both scientific and regulatory reasons. Genomic approaches offer high resolution identification of strains by comparing those with other well-characterized, safe, and efficacious probiotic strains. Comparative genomics studies further help to understand the molecular basis of probiotic efficacy, as well as the survival and adaptation of these probiotic strains in the gastrointestinal tract. Most importantly, genomic analyses allow for rapid screening of probiotic candidates for genes encoding antimicrobial resistance, virulence factors, toxins, and biogenic amines, facilitating better understanding of the safety of the probiotic strain of interest. Finally, genome-based analyses also help to investigate the stability of probiotic strains.

The goal of this study was to provide *in silico*, *in vitro* and *in vivo* evidence to support the safety of *L*. *reuteri* ATCC PTA-126787 & ATCC PTA-126788 (hereafter referred to as PTA-126787 and PTA-126788) for their use as probiotics in humans as well as animals. More specifically, the strains were identified using a combination of biochemical, 16S rRNA and whole-genome sequencing analyses. The genomes were screened for potential genes encoding antimicrobial resistance, toxins, virulence factors and other harmful metabolites. *In silico* data were further confirmed using *in vitro* experiments. The strains were finally analysed for safety using the broiler chicken as an *in vivo* model.

## Materials and methods

### Ethics statement

All chickens were housed and cared for under the Guide for the Care and Use of Agricultural Animals in Research and Teaching and all local standard operating procedures. The study was reviewed and approved by the Animal Care and Use Committee of the institution performing the study (Assigned ACUP# 1399).

### Bacterial strains and culture conditions

The *L*. *reuteri* strains described in this study were routinely propagated on *Lactobacilli* de Man Rogosa Sharpe (MRS, BD Difco) medium anaerobically at 37°C. *L*. *reuteri* strain DSM 17938 was used as a reference strain for biochemical identification, D- and L-lactate production, autoaggregation, resistance to bile salts and acidic pH assays. *L*. *reuteri* strain ATCC 23272 was used as a reference strain for growth kinetics, autoaggregation, biogenic amine production and D- and L-lactate production assays. *L*. *acidophilus* strain ATCC 4356 was used as a reference strain for growth kinetics assay.

### Molecular identification

The strains were identified using 16S rRNA sequencing. Briefly, *L*. *reuteri* strains were grown in *Lactobacilli* MRS broth overnight for 14–16 hours under anaerobic conditions at 37°C. One hundred microliters of the culture were pelleted by centrifugation and resuspended in 50 μL of nuclease-free water. The resuspended culture was heated at 98°C for 10 minutes. The debris was pelleted by brief centrifugation and the supernatant was used as a template for PCR. The 16S rRNA gene was amplified by PCR using 3 μL of the DNA template and universal primers 16S rRNA gene F, 5’-AGAGTTTGATCCTGGCTCAG-3’ and 16S rRNA gene R, 5’-CTTGTGCGGGCCCCCGTCAATTC-3’. The amplicons were PCR purified using the QIAquick PCR Purification Kit (Qiagen Inc.) following manufacturer’s instructions and sequenced by Sanger sequencing by GenScript. The sequences were then searched against the NCBI nucleotide collection (nr/nt) database using the BLAST algorithm.

### Biochemical identification

The strains were profiled for enzymatic activity and carbohydrate fermentation using API 50 CHL strips (bioMérieux), following the manufacturer’s instructions. The *L*. *reuteri* strain DSM 17938 was used as a positive control.

### Enzyme profiling

The enzymatic profiles of *L*. *reuteri* strains were determined using the APIZym test strips (bioMérieux), following manufacturer’s instructions.

### Growth kinetics

The *L*. *reuteri* strains were grown in MRS broth overnight for 14–16 hours under anaerobic conditions at 37°C. The next morning, the cultures were adjusted to an OD_600_ of 0.1 and monitored for growth by plating on *Lactobacilli* MRS agar at 0, 1, 2, 4, and 8 hours. Human *L*. *reuteri* strain 23272 and *L*. *acidophilus* strain ATCC 4356 were used as controls.

### Isolation of high molecular weight DNA

High molecular weight DNA for PacBio sequencing was isolated using the phenol:chloroform method. Briefly, *L*. *reuteri* strains were grown in *Lactobacilli* MRS broth overnight under anaerobic conditions for 14–16 hours. The cells were harvested by centrifugation at 4,000*g* RCF for 10 minutes at 4°C. The pellet was washed once by 1 mL of TE buffer (10 mM Tris-HCl and 1 mM EDTA, pH 8.0) and resuspended in 0.5 mL of TE containing 1.2% Triton X-100 and 10 mg/mL of lysozyme (Sigma Aldrich) and incubated at 37°C for 1 hour. After incubation, 20 μL of proteinase K was added, mixed several times, and incubated at 55°C for 1 hour. Twenty microliters of RNase was then added and incubated at 37°C for an additional 30 minutes. Approximately, 600 μL of phenol:chloroform:isoamyl alcohol (25:24:1; ThermoFisher Scientific) mixture (pH 8.0) was added, the tubes were inverted several times and centrifuged at 11,200 × *g* for 10 minutes at 4°C. The upper aqueous phase was carefully transferred to a new 1.5 mL centrifuge tube. The above phenol:chloroform step was repeated one more time. 0.5 mL of chloroform was added, the tubes were inverted several times and centrifuged at 11,200*g* RCF for 10 minutes. The upper aqueous phase was carefully transferred to a new centrifuge tube. 0.45 mL of isopropanol was layered onto the aqueous phase containing genomic DNA and the tubes were gently shaken to precipitate high molecular weight genomic DNA. The precipitated DNA was removed with a sterile loop and transferred to a new tube containing 1 mL 70% ethanol. The tubes were centrifuged at 11,200*g* RCF for 5 minutes at 4°C. The DNA pellet was briefly air-dried and 300 μL of nuclease free water was added, and then allowed to dissolve overnight at 4°C. The dissolved DNA was gently mixed with a big-bore tip and stored at -20°C. The isolated DNA was analyzed for quantity using Qubit (Thermo Fisher Scientific, Inc.).

### Whole genome sequencing and assembly

The bacterial genomic DNA samples were shipped on dry-ice to DNA Link, Inc (San Diego, CA; https://www.dnalink.com/english/) for whole genome sequencing using PacBio RSII platform. Briefly, 20 kb DNA fragments were generated by shearing genomic DNA using the Covaris G-tube according to the manufacturer’s recommended protocol (Covaris). Smaller fragments were purified by the AMpureXP bead purification system (Beckman Coulter). For library preparation, 5μg of genomic DNA was used. The SMRTbell library was constructed using SMRTbell™ Template Prep Kit 1.0 (PacBio^®^). Small fragments were removed using the BluePippin Size selection system (Sage Science). The remaining DNA sample was used for large-insert library preparation. A sequencing primer was annealed to the SMRTbell template and DNA polymerase was bound to the complex using DNA/Polymerase Binding kit P6 (PacBio^®^). Following the polymerase binding reaction, the MagBead was bound to the library complex with MagBeads Kit (PacBio^®^). This polymerase-SMRTbell-adaptor complex was loaded into zero-mode waveguides. The SMRTbell library was sequenced by 2 PacBio^®^ SMRT cells (PacBio^®^) using the DNA sequencing kit 4.0 with C4 chemistry (PacBio^®^). A 1×240-minute movie was captured for each SMRT cell using the PacBio^®^ RS sequencing platform. The genome was further assembled by DNA link, Inc with HGAP.3 protocol.

### Genome annotation and feature prediction

Genome annotation was carried out using a custom annotation pipeline by combining several prediction tools. Coding sequences, transfer RNA and transmembrane RNA were predicted and annotated using Prokka v 1.14.5 [36–38]. Ribosomal binding site (RBS) prediction was carried out using RBSFinder [39]. TranstermHP v2.08 was used to predict Rho-independent transcription terminators (TTS) [40]. Ribosomal RNA and other functional RNAs such as riboswitches and non-coding RNA was annotated with Infernal v1.1.2 [41]. Operons were predicted based on primary genome sequence information with Rockhopper v2.0.3 using default parameters [42].

### Data deposition

The raw sequencing reads, genome assemblies and annotations in this study were deposited in the NCBI BioProject under project PRJNA675717.

Accession numbers:

**Table:** 

Serial No.	Sample	BioSample	Accession number	SRA number
1.	ATCC PTA-126787	SAMN16712075	CP065330-CP065334	SRX9689306
2.	ATCC PTA-126788	SAMN16712076	CP065849-CP065855	SRX9689307

### Phylogenetic analyses

Phylogenetic relationships of the genomes were explored with UBCG v3.0 using default settings [43]. This software tool employs a set of 92 single-copy core genes commonly present in all bacterial genomes. These genes then were aligned and concatenated within UBCG using default parameters. The estimation of robustness of the nodes is done through the gene support index (GSI), defined as the number of individual gene trees, out of the total genes used, that present the same node. A maximum-likelihood phylogenetic tree was inferred using FastTree v.2.1.10 with the GTR+CAT model [44].

### Comparative genomic analyses

OrthoFinder v2.3.11 [45] was used to determine orthologous relationships between protein sequences inferred from PTA-126787 and PTA-126788 with protein sequences of strains ATCC 53608, CF48-3A, DSM20016 and SD2112 (the parent strain of DSM17938) downloaded from GenBank [46]. Pairwise Average Nucleotide Identities (ANI) values were calculated all-against-all, using FastANI v 1.32 [47].

### Identification of prophages, transposases and other insertion sequences (IS)

Insertion sequence prediction was done using ISEscan v.1.7.2.1 [48]. Prophage prediction was done using PhiSpy v4.2.6 which combines similarity‐ and composition‐based strategies [49].

### Identification of CRISPR-Cas sequences

Coding sequences for Clustered Regularly Interspaced Short Palindromic Repeats (CRISPR) and CRISPR-associated genes (Cas) were searched using CRISPRDetect version 2.2 [50]. However, no CRISPR sequences were identified in both the genomes.

### Identification of virulence determinants and antimicrobial resistance genes

Protein-encoding genes related to virulence were searched manually based on functional annotation of the genomes. Automated screening of whole genome sequences of both strains against the Virulence Factor Database (VFDB), a comprehensive repository of known bacterial virulence factors and other putative adverse metabolites [51], ARG-ANNOT [52], ResFinder [53] and NCBI-AMR databases (2020-Jun-15) was performed using Abricate version 0.9.9 [54].

### Identification of genes encoding toxic metabolites

Analysis was performed on the genomes manually to identify homologs of histidine decarboxylase, tyrosine decarboxylase, lysine decarboxylase, ornithine decarboxylase, agmatine deiminase, agmatine::putrescine antiporter, multicopper oxidase and other potential genes involved in the production of biogenic amines.

### Genes involved in lactic acid production and other beneficial metabolites

Sequences encoding putative genes involved in lactic acid production and other metabolites were identified by manual search of functional annotations.

### Antimicrobial susceptibility profiling

Antimicrobial susceptibility testing was performed using broth microdilution method, using LSB medium (Mueller Hinton broth containing 5% horse blood) following Clinical and Laboratory Standards Institute (CLSI, 28^th^ edition) guidelines. Two-fold dilutions of the clinically relevant antibiotics (Clindamycin, Chloramphenicol, Erythromycin, Gentamicin, Kanamycin, Streptomycin, Tetracycline and Ampicillin, all purchased from Sigma Aldrich) were prepared in LSB medium. Approximately, 50 μL of 1 × 10^5^ CFU/mL of the *L*. *reuteri* cells were added into each well. “No antibiotic” and “medium” alone controls were included. *Escherichia coli* ATCC 25923, *Pseudomonas aeruginosa* ATCC 27853, *Staphylococcus aureus* ATCC 29213, *Enterococcus faecalis* ATCC 29212, *Streptococcus pneumonia* ATCC 49619 and *Lacticaseibacillus paracasei* ATCC 334 were used as quality control organisms. The plates were incubated for 24–48 hours under microaerophilic conditions. Minimum inhibitory concentration (MIC) was defined as the lowest concentration of antibiotic that showed complete inhibition of *L*. *reuteri* growth. The strains were classified as susceptible or resistant using the microbiological cut offs established by EFSA [35].

### Biogenic amine production

The ability of *L*. *reuteri* strains to produce biogenic amines was determined as previously described [55]. Briefly, *L*. *reuteri* cultures were grown in MRS broth supplemented with L-tyrosine (0.1% m/v), L-histidine (0.1% m/v), L-arginine (0.1% m/v) or L-lysine (0.1% m/v) and pyridoxal-5-phosphate (0.005% m/v) under anaerobic conditions at 37°C overnight. The cultures were then plated on supplemented decarboxylase broth base as described by Bover-Cid and Holzapfel [56] and colour development was recorded after 48 hours of incubation under anaerobic conditions at 37°C.

### D- and L-lactate production

The amounts of D- and L-lactate produced were quantified using D-/L-Lactic Acid (D-/L-Lactate) (Rapid) Assay Kit (Megazyme), following manufacturer’s instructions. Briefly, *L*. *reuteri* strains were grown in MRS broth incubated at 37°C for 14–16 hours. The cultures were centrifuged at 4,000*g* RCF for 10 minutes at 4°C and the supernatant was collected into a 1.5 mL Eppendorf tube and filter sterilized. 1.0 mL of the filter sterilized supernatant was used for lactic acid quantification as described in the manual provided by the manufacturer.

### Autoaggregation

The ability of *L*. *reuteri* strains to autoaggregate was assayed as follows. *L*. *reuteri* strains were grown in MRS broth overnight for 14–16 hours under anaerobic conditions at 37°C. The cultures were adjusted to an OD_600_ of 0.1 and allowed to grow for another 14–16 hours and observed for aggregate formation. Autoaggregation was quantified as described previously with some minor modifications [57]. *L*. *reuteri* strains were grown in MRS broth overnight for 14–16 hours under anaerobic conditions at 37°C. The cultures were washed twice with PBS (pH 7.2) by centrifuging at 11,200*g* RCF for 10 minutes at 4°C. The washed cell pellets were then resuspended in PBS (pH 7.2) and adjusted to an OD_600_ of 0.5 to standardize the number of bacterial cells (10^7^−10^8^ CFU/ml). The suspensions were incubated as 1 ml aliquots under anaerobic conditions at 37°C for 5 hours. The OD_600_ was recorded after 5 hours. Autoaggregation percentage was calculated as follows: [1- (Absorbance at 5 hours/ Absorbance at 0 hour)] x 100.

### Hydrogen peroxide production

The ability of *L*. *reuteri* strains to produce hydrogen peroxide was assessed as previously described [58]. Briefly, MRS agar plates were prepared with 0.25 mg/mL of tetramethylbenzidine and 0.01 mg/mL of horseradish peroxidase. *L*. *reuteri* strains were streaked on the supplemented MRS agar plates and incubated for 24 hours and coloration of the colonies/culture was recorded. White bacterial colonies/culture indicates no hydrogen peroxide production, pale blue colonies/culture indicates poor production and dark blue colonies/culture indicates high production.

### Resistance to bile salts

The ability of cultures to tolerate bile salts was determined as follows: *L*. *reuteri* strains were grown under anaerobic conditions for 14–16 hours at 37°C. The culture was inoculated into fresh MRS broth (pH adjusted to 6.4, optimal pH for *L*. *reuteri* growth) containing 0.3% bile salts (Oxoid, USA) at a rate of 1% inoculum and incubated under anaerobic conditions at 37°C for 4 hours. Samples were collected at 0 and 4 hours after incubation and analysed for CFU counts.

### Resistance to acidic pH

The tolerance of *L*. *reuteri* strains to low pH was determined as described below. *L*. *reuteri* strains were grown under anaerobic conditions for 14–16 hours at 37°C. The cells were harvested by centrifugation at 4,000*g* RCF for 10 minutes at 4°C and the pellet was resuspended in sterile PBS (adjusted to a pH of 2.5) to an OD_600_ of 0.5. The cultures were then incubated at 37°C for 3 hours under anaerobic conditions. Aliquots were collected at time 0 and 3 hours, respectively after incubation, serially diluted in PBS and plated on MRS to determine the CFU counts.

### *In vivo* safety assessment

The safety of *L*. *reuteri* strains was tested using Specific Pathogen Free (SPF) broiler chickens. Forty-two White Leghorn, mixed sex chicks of day-old age were purchased from Valo BioMedia. At arrival, the chicks were tagged via wing web. The birds were fed commercially available non-medicated feed *ad libitum*. Briefly, day-old chicks were randomly grouped into three groups with 14 birds in each group. Group 1 was administered with 1 × 10^7^ CFU/bird/day of *L*. *reuteri* PTA-126787 in drinking water from day 1 to day 26. Group 2 was administered with 1 × 10^7^ CFU/bird/day of *L*. *reuteri* PTA-126788 in drinking water from day 1 to day 26. Group 3 served as no treatment control. The birds were examined for adverse events, morbidity, and mortality on a daily basis. On day 31, the birds were euthanized, and observed for any gross lesions, indicative of health issues. More specifically, lungs, trachea, liver, spleen, kidneys, intestine were observed for gross lesions and scored as normal or abnormal. Gut (2 cm of cecal-rectal junction), lung (dime size) and tracheal (2 cm long) samples were collected from 5 birds per group in buffered formalin, analysed for histopathology and scored as described in **S1 File**.

## Results

### *L*. *reuteri* isolation and molecular identification

**Fig 1:**
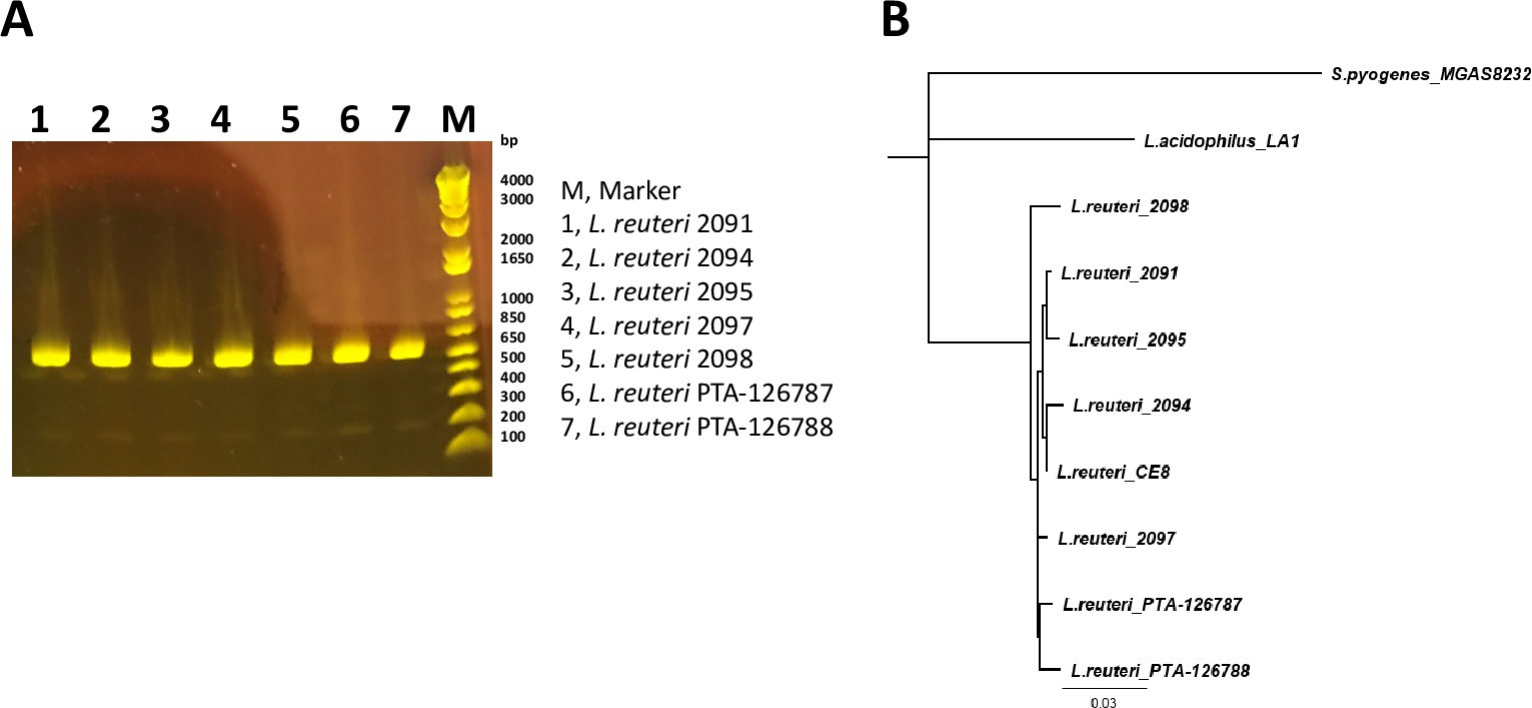
Identification of *L*. *reuteri* strains by 16S rRNA amplicon sequencing. *L*. *reuteri* strains were identified by PCR amplification and sequencing of the 16S rRNA variable region. **A**. Agarose gel electrophoresis of the 16S rRNA PCR product. **B**. Phylogenetic analysis of the 16S rRNA sequence along with other *L*. *reuteri* sequences. *Streptococcus pyogenes* was included as an outgroup.

A library of seven *L*. *reuter*i strains along with the two strains described in this study were isolated from the cecum of older broiler chickens at Elanco Animal Health, Cuxhaven, (Germany). Based on the 16S rRNA amplicon sequencing and respective BLAST search comparison results, all the seven strains, including PTA-126787 and PTA-126788 showed closest homology to published *L*. *reuteri* sequences, suggesting that our strains belong to the *L*. *reuteri* species **(Fig 1A and 1B)**.

### Biochemical identification

When tested with API 50 CHL, the final two *L*. *reuteri c*andidates, PTA-126787 and PTA-126788, were identified as *Limosilactobacillus fermentum* (previously *Lactobacillus fermentum)* with 92.3% identity (**Table 1**). The positive control *L*. *reuteri* DSM 17938 was also identified as *L*. *fermentum* with 92.3% identity (**Table 1**). The fermentation profile of *L*. *reuteri* is similar to that of *L*. *fermentum* and the APIweb^TM^ software version 5.0 does not have the capability to distinguish between the 2 species.

**Table 1:** Carbohydrate fermentation profile of *L*. *reuteri* strains PTA-126787 and PTA-126788 by API 50 CHL.

Substrate	PTA-126787	PTA-126788	DSM 17938	Substrate	PTA-126787	PTA-126788	DSM 17938
Negative control	-	-	-	Esculin ferric citrate	+	+	+
Glycerol	-	-	-	Salicin	-	-	-
Erythritol	-	-	-	D-Cellobiose	-	-	-
D-Arabinose	-	-	-	D-Maltose	+	+	+
L-Arabinose	+	+	+	D-Lactose	+	+	+
D-Ribose	+	+	+	D-Melibiose	+	+	+
D-Xylose	-	-	-	D-Saccharose	+	+	+
L-Xylose	-	-	-	D-Trehalose	-	-	-
D-Adonitol	-	-	-	Inulin	-	-	-
Methyl-βD-xylopyranoside	-	-	-	D-Melezitose	-	-	-
D-Galactose	+	+	+	D-Raffinose	+	+	+
D-Glucose	+	+	+	Amidon	-	-	-
D-Fructose	-	-	-	Glycogen	-	-	-
D-Mannose	-	-	-	Xylitol	-	-	-
L-Sorbose	-	-	-	Gentibiose	-	-	-
L-Rhamnose	-	-	-	D-Turanose	-	-	-
Dulcitol	-	-	-	D-Lyxose	-	-	-
Inositol	-	-	-	D-Tagatose	-	-	-
D-Mannitol	-	-	-	D-Fucose	-	-	-
D-Sorbitol	-	-	-	L-Fucose	-	-	-
Methyl-αD-mannopyroside	-	-	-	D-Arabitol	-	-	-
Methyl-αD-glucopyranoside	-	-	-	L-Arabitol	-	-	-
N-Acetylglucosamine	-	-	-	Potassium gluconate	+	+	+
Amygdalin	-	-	-	Potassium 2-ketogluconate	-	-	-
Arbutin	-	-	-	Potassium 5-ketogluconate	-	-	-

+, positive reaction; -, negative reaction

### Enzyme profile

Enzyme profile is a good indicator of both the probiotic function as well as safety. APIZym test is a rapid semiquantitative assay to detect 19 enzymatic reactions. Unlike API 50 CHL, no databases exist to identify bacteria based on APIZym profiles. As shown in **Table 2**, both *L*. *reuteri* strains showed similar enzyme profiles to that of *L*. *reuteri* strain DSM 17938. The two strains showed strong leucine arylamidase, valine arylamidase, acid phosphatase, α-galactosidase and β-galactosidase activities, while both were negative for alkaline phosphatase, lipase, trypsin, α-chymotrypsin, β-glucosidase, α-mannosidase and α-fucosidase activities. In general, the enzymatic reactions from APIZym testing were in good agreement with carbohydrate fermentation by API 50 CHL.

**Table 2:** Enzymatic profiles of *L*. *reuteri* strains PTA-126787 and PTA-126788 by APIZym.

Enzyme assayed	Substrate	PTA-126787	PTA-126788	DSM 17938
Alkaline phosphatase	2-naphthyl phosphate	-	-	-
Esterase (C 4)	2-naphthyl butyrate	+	+	+
Esterase Lipase (C 8)	2-naphthyl capylate	+/-	+/-	-
Lipase (C 14)	2-naphthyl myristate	-	-	-
Leucine arylamidase	L-leucyl-2-naphthylamide	+++	+++	++
Valine arylamidase	L-valyl-2-naphthylamide	++	++	+
Cystine arylamidase	L-cystyl-2-naphthylamide	+	+	+
Trypsin	N-benzoyl-DL-argine-2-naphthylamide	-	-	-
α-chymotrypsin	N-glutaryl-phenylalanine-2-naphthylamide	-	-	-
Acid phosphatase	2-naphthyl phosphate	+++	+++	++
Naphthol-AS BI-phosphohydrolase	Naphthol-AS-BI-phosphate	+	+	-
α-galactosidase	6-Br-2-naphthyl-αD-galactopyranoside	+++	+++	+++
β-galactosidase	2-naphthyl-βD-galactopyranoside	+++	+++	+++
β-glucuronidase	Naphthol-AS-BI-βD-glucuronide	+	+	+
α-glucosidase	2-naphthyl-αD-glucopyranoside	+	+	+
β-glucosidase	6-Br-2-naphthyl-βD-glucopyranoside	-	-	-
N-acetyl-β-glucosaminidase	1-naphthyl-N-acetyl-βD-glucosaminide	+/-	+/-	-
α-mannosidase	6-Br-2-naphthyl-αD-mannopyranoside	-	-	-
α-fucosidase	2-naphthyl-αL-fucopyranoside	-	-	-

+++, very strong positive enzymatic reaction; ++, strong positive enzymatic reaction; +, positive enzymatic reaction; -, negative enzymatic reaction; +/-, inconclusive

### Growth profiles

**Fig 2:**
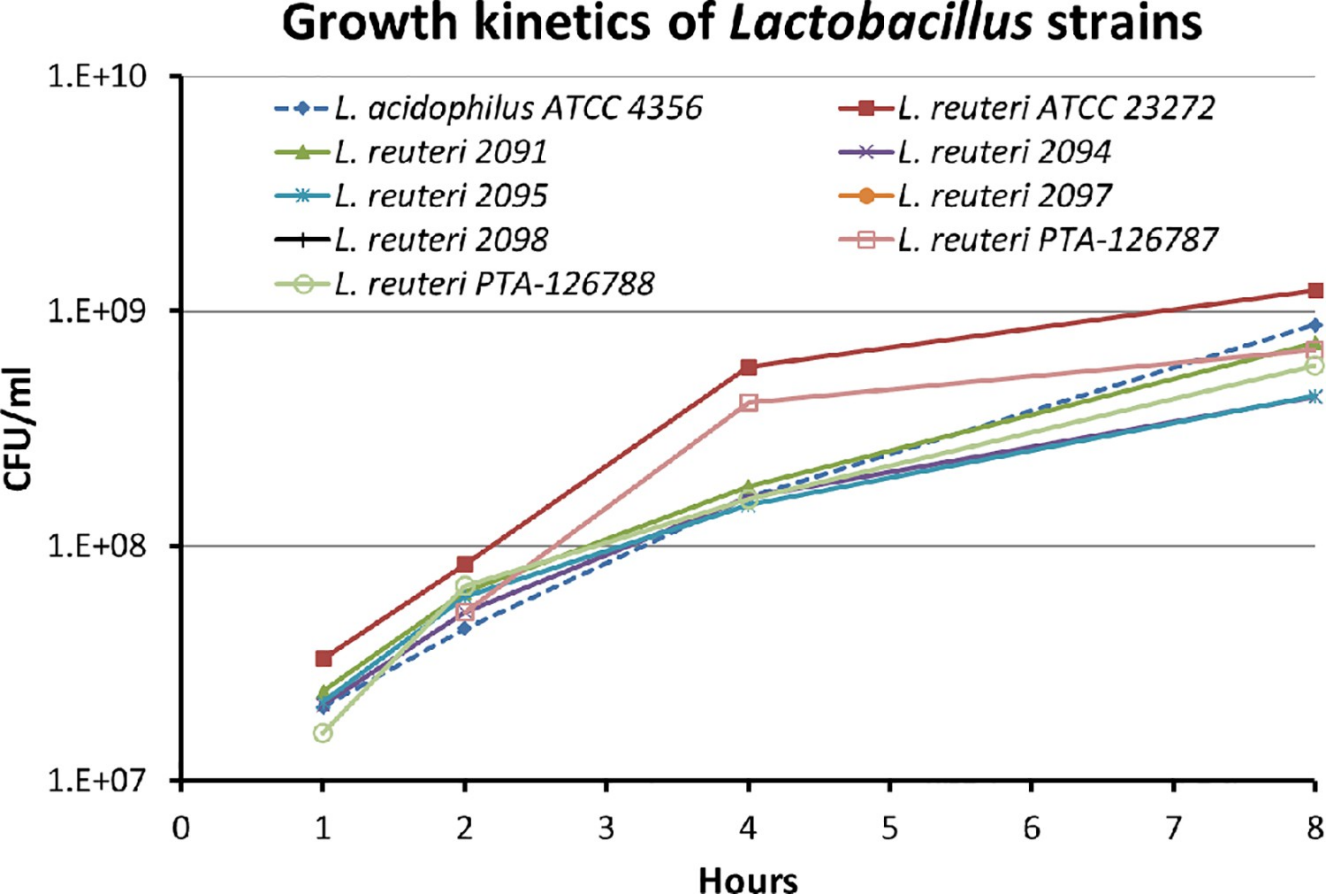
Growth profiles of *L*. *reuteri* strains in MRS broth. Growth profiles were assessed by growing the strains in MRS broth and determining the CFU counts at different time points. The data shown is representative of 3 independent experiments.

All the *L*. *reuteri* strains had similar growth profiles, including PTA-126787 and PTA-126788 (**Fig 2**), and the profiles were comparable to that of human *L*. *reuteri* strain ATCC 23272 and *Lactobacillus acidophilus strain* ATCC 4356.

### *In silico* analyses

#### A. Genomic characterization

**Fig 3:**
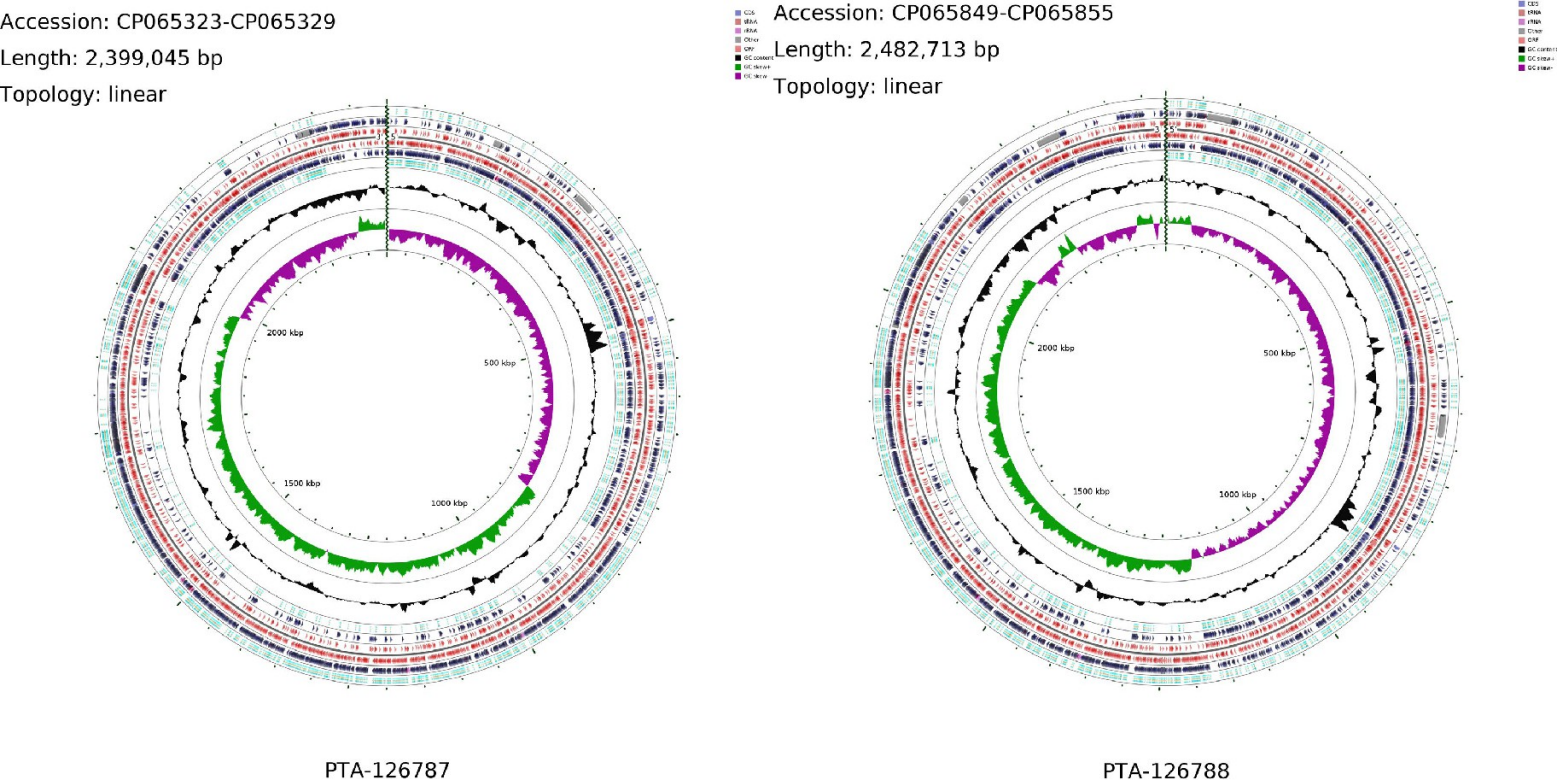
Chromosomal map of *L*. *reuteri* strains PTA-126787 and PTA-126788. The concentric circles show, reading outwards: GC skew, GC content, AT skew, AT content, COG classification of proteins, CDS on reverse strand, ORFs on three frames in reverse strand, ORFs on three frames in forward strand, CDS on forward strand and COG classification of proteins on forward strand.

The genomes of *L*. *reuteri* strains PTA-126787 and PTA-126788 were sequenced by PacBio sequencing platform. Strain PTA-126787 contains 5 contigs yielding a total estimated genome size of 2.4 Mb and strain PTA-126788 contains 7 contigs yielding an estimated genome size of 2.4 Mb. The genome properties, prediction and annotation of different features are summarized in Table 3. The circular representation of the complete genomes of both strains is shown in Fig 3. The whole-genome sequencing project was deposited at DDBJ/ENA/GenBank under BioProject number PRJNA675717.

**Table 3:** Genomic properties of *L*. *reuteri* strains PTA-126787 and PTA-126788.

Feature	PTA-126787	PTA-126788
Contigs	5	7
Coding sequence	2427	2495
Prophages	6	8
Mobile Element	86	88
Non-coding RNA	21	17
Operons	501	541
Ribosomal RNA	18	17
Ribosomal binding site	2359	2396
Transcription terminator	1182	1241
Riboswitch	26	25
Transfer RNA	73	74
Transfer-messenger RNA	1	1

#### B. Phylogenetic analysis

**Fig 4:**
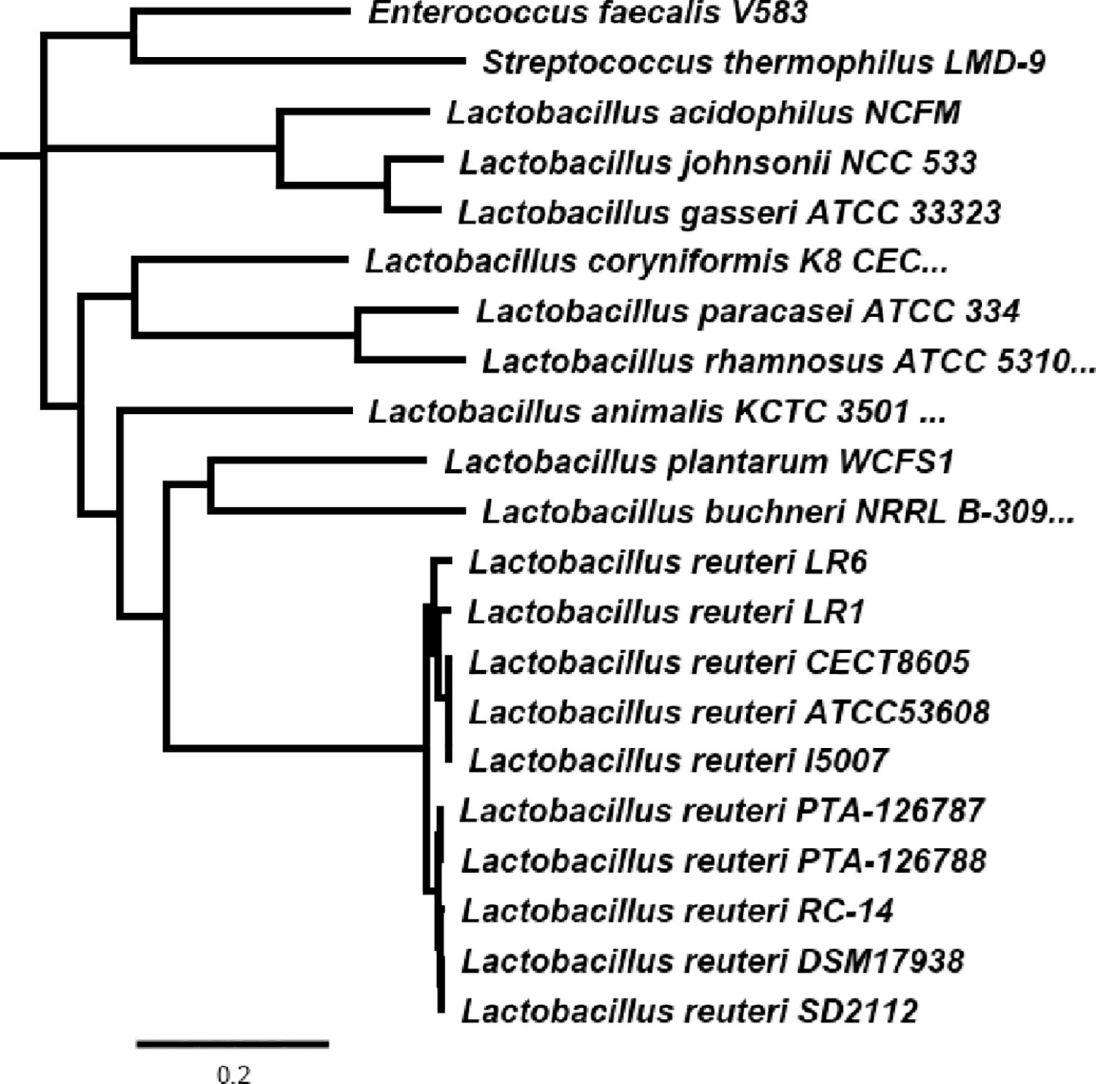
Phylogenetic relationship of *L*. *reuteri* strains PTA-126788 and PTA-126787 to other known human *L*. *reuteri* strains using 92 core genes. The phylogenetic relationship was explored using UBCG v3.0 and a maximum likelihood tree was inferred using GTR+CAT model. *Streptococcus thermophilus* and *Enterococcus faecalis* were used as outgroups.

Phylogenetic relationships of the genomes were explored with UBCG v3.0 which employs a set of 92 single-copy core genes commonly present in all bacterial genomes. These genes then were aligned and concatenated within UBCG using default parameters. The estimation of robustness of the nodes is done through the gene support index (GSI), defined as the number of individual gene trees, out of the total genes used, that present the same node. As shown in **Fig 4**, both strains PTA-126787 and PTA-126788 showed closest relationship to *L*. *reuteri*. Average Nucleotide Identities were calculated between closely related genomes and is shown in **Table 4**.

**Table 4:** Average Nucleotide Identity (ANI) of *L*. *reuteri* PTA-126787 and PTA-126788 with closely related human probiotic strains.

Query genome	Reference genome	%ANI	Orthologous matches	Sequence fragments
PTA-126787	CF48-3A	98.1	560	797
PTA-126787	RC-14	98.0	537	797
PTA-126787	RC-18	98.0	537	797
PTA-126787	SD2112	98.0	590	797
PTA-126787	DSM17938	98.0	591	797
PTA-126787	DSM20016	95.4	523	797
PTA-126787	ATCC53608	95.1	532	797
PTA-126788	CF48-3A	98.2	553	825
PTA-126788	SD2112	98.1	581	825
PTA-126788	DSM17938	98.1	582	825
PTA-126788	RC-14	98.0	532	825
PTA-126788	RC-18	98.0	532	825
PTA-126788	DSM20016	95.6	516	825
PTA-126788	ATCC53608	95.0	545	825

#### C. Comparative genomics analyses

Ortholog analysis was performed to identify paralogous and/or orthologous relationships between genomes of *L*. *reuteri* strains PTA-126787 and PTA-126788 against *L*. *reuteri* strains ATCC 53608, CF48-3A, DSM20016 and SD2112 (the parent strain of DSM17938) using OrthoFinder (**S1 and S2 Tables**). Genes unique to strains PTA-126787 and PTA-126788 are presented in **S3 Table**. *L*. *reuteri* strains PTA-126787 and PTA-126788 shared the highest number of orthologs amongst the strains compared in the analysis with 2264 and 2242 shared genes among them, respectively (**S1 Table**).

#### D. Screening for prophages, insertion sequences and transposases

**Fig 5:**
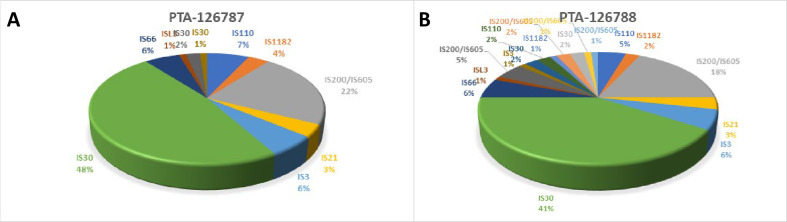
Distribution of predicted IS family within the genomes of *Lactobacillus reuteri* strains PTA-126787 (A) and PTA-126788 (B) using ISEScan.

Both strains were scanned for the presence of mobile genetic elements such as prophages, insertion sequences (IS) and transposases. Six prophage regions in strain PTA-126787 and eight regions in PTA-126788 were identified (**S1 Fig**). However, there were 12 phage genes (all coding for Tyrosine recombinase protein) in PTA-126788 that were outside of prophage regions. Putative IS and associated proteins predicted by ISEscan reveal 86 coding sequences in 10 IS families in strain PTA-126787 and 88 coding sequences in 18 IS families in strain PTA-126788 (**Fig 5; S4 Table**).

#### E. Absence of virulence factors and toxins

Both *L*. *reuteri* PTA-126787 (5 contigs) and PTA-126788 (7 contigs) strains were confirmed to be free of known virulence factors and/or toxins by comparing against virulence factor database (VFDB; search parameters of ≥80% identity and ≥80% alignment length/coverage), which is an integrated comprehensive online resource database for curating information about bacterial virulence factors and/or toxins [51].

#### F. Absence of acquired antimicrobial resistance genes

The Pariza *et al*. [34] decision tree and the EFSA Panel on Additives and Products or Substances used in Animal Feed [35] recommend that microbial strains used in food applications must not harbor acquired antimicrobial resistance genes to clinically relevant antimicrobials. Search for antimicrobial resistance genes was carried out for both *L*. *reuteri* strains by comparing the genomes against multiple AMR databases including NCBI-AMR, Resfinder DB and ARG-ANNOT using Abricate. The screening identified tetracycline-resistant ribosomal protection protein (*tetW*) that confers resistance to tetracycline as one potential gene of health concern (**S5 Table**) [34].

#### G. Screening for genes involved in biogenic amines and toxins

Functional annotation of the entire genomes of *L*. *reuteri* strains PTA-126787 and PTA-126788 revealed that they do not contain any known protein-encoding genes involved in the production of biogenic amines with the exception of CDS encoding for arginine deiminase. No other toxins were identified (**S6 Table**).

#### H. Genes involved in the production of lactic acid and other beneficial metabolites

Both strains, PTA-126787 and PTA-126788, contain genes responsible for production of lactic acids. A total of four coding sequences (CDS) were predicted to encode for D-lactate dehydrogenase (EC 1.1.1.28) and four CDS for L-lactate dehydrogenase (EC 1.1.1.27) were found on different loci within the genome (**S7 Table**). However, IVR12_00498 gene in strain PTA-126788 is a pseudogene due to a frameshift mutation. The coding sequence putative for a therapeutically useful peptide, S-ribosylhomocysteinelyase (EC 4.4.1.21; IU404_00512 and IVR12_00964) was also present in the genomes of strains PTA-126787 and PTA-126788, respectively.

Several coding sequences involved in adhesion of *Lactobacilli* to intestinal epithelium were identified in the genome (**S8 Table**). Some of the genes involved in adhesion to host found in both strains are sortase A, epsilon subunit related 3’-5’ exonuclease, exopolysaccharide biosynthesis protein and ATP synthase epsilon subunit (**S8 Table**). Search for desired stress tolerance features in both *L*. *reuteri* strains revealed the presence of CDS predictably encoding for DNA protection during starvation protein (**S8 Table**). Another stress resistant gene putatively encoding for Phosphate starvation-inducible PhoH-like protein, predicted ATPase was also found in both strains (**S8 Table**).

### Antimicrobial susceptibility

Minimum inhibitory concentrations were analyzed against relevant antibiotics according to EFSA guidelines (EFSA Panel on Additives and Products or Substances used in Animal Feed) [35], including Ampicillin, Vancomycin, Gentamicin, Kanamycin, Streptomycin, Erythromycin, Clindamycin, Tetracycline and Chloramphenicol. *L*. *reuteri* PTA-126788 and PTA-126787 strains were determined to be sensitive to all relevant tested antibiotics according to EFSA guidelines [35], with MIC values at or below the reported species characteristic cut-off values (**Table 5**), except for tetracycline. For tetracycline, the MIC values for our strains were two-fold dilution above the EFSA microbiological cut off value, in one of the two biological replicates. However, this is considered acceptable due to the technical variation of the phenotypic method as recognized previously [59].

**Table 5:** Susceptibility of *L*. *reuteri* PTA-126787 and PTA-126788 to EFSA critically important antibiotics.

	*L*. *reuteri* PTA-126788	*L*. *reuteri* PTA-126787	EFSA microbiological cut off values for *L*. *reuteri*
**Clindamycin**	≤0.06	≤0.06	4
**Chloramphenicol**	2	2	4
**Erythromycin**	0.12	0.12	1
**Gentamicin**	1	1	8
**Kanamycin**	16	16	64
**Streptomycin**	8	8	64
**Tetracycline**	32/64	32/64	32
**Ampicillin**	1	1	2

### Biogenic amine production

Many lactic acid bacteria produce biogenic amines such as histamine, tyramine, putrescine and/or cadaverine by amino acid decarboxylation of histidine, tyrosine, ornithine and/or lysine, respectively. The few instances of toxicity cases are associated with histamine and to some extent tyramine. Consistent with the bioinformatics results, neither of the subject *L*. *reuteri* strains were able to produce the major biogenic amines histamine, tyramine, putrescine or cadaverine. As expected, *L*. *reuteri* ATCC 23272 produced a positive reaction in the area of bacterial growth on the decarboxylase base media supplemented with L-histidine. Control plates lacking these amino acids showed no positive reaction for any of the strains tested.

### D- and L-Lactate production

**Fig 6:**
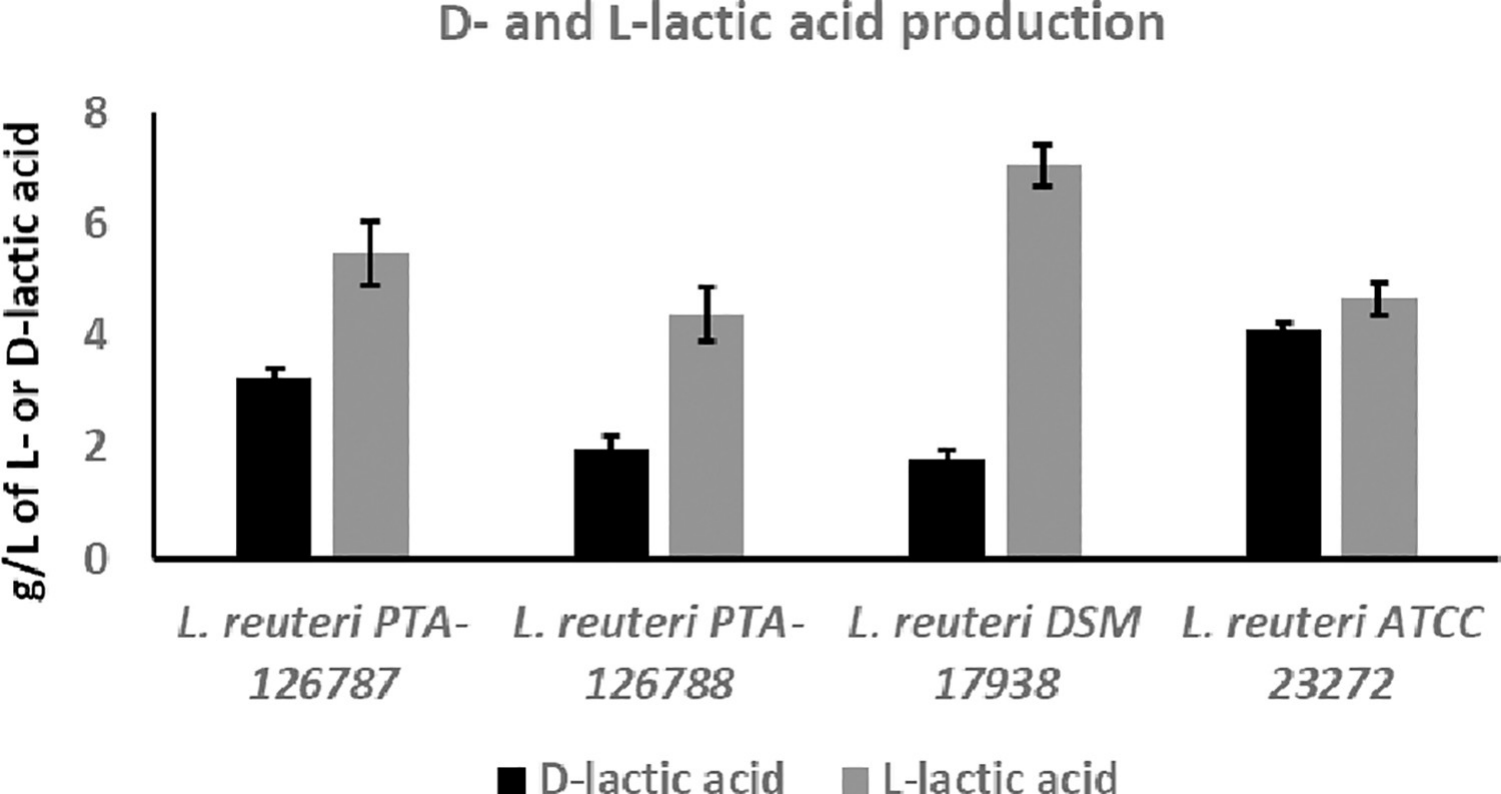
Production of D- and L-lactic acid by *L*. *reuteri* strains. L- and D-lactic acids were quantified using D-/L-lactic acid (D-L-lactate) Rapid Assay Kit (Megazyme). The data represent the mean ± SD from 3 independent experiments.

Quantitative determination of lactic acid production showed that the two *L*. *reuteri* strains produce both L- and D-lactic acids but predominantly L-lactic acid (**Fig 6**). Similarly, *L*. *reuteri* DSM 17938 also produced L- and D-lactic acids but predominantly L-lactic acid (**Fig 6**). *L*. *reuteri* strain ATCC 23272 produced approximately equal amounts of L- and D-lactic acids (**Fig 6**).

### Autoaggregation

**Fig 7:**
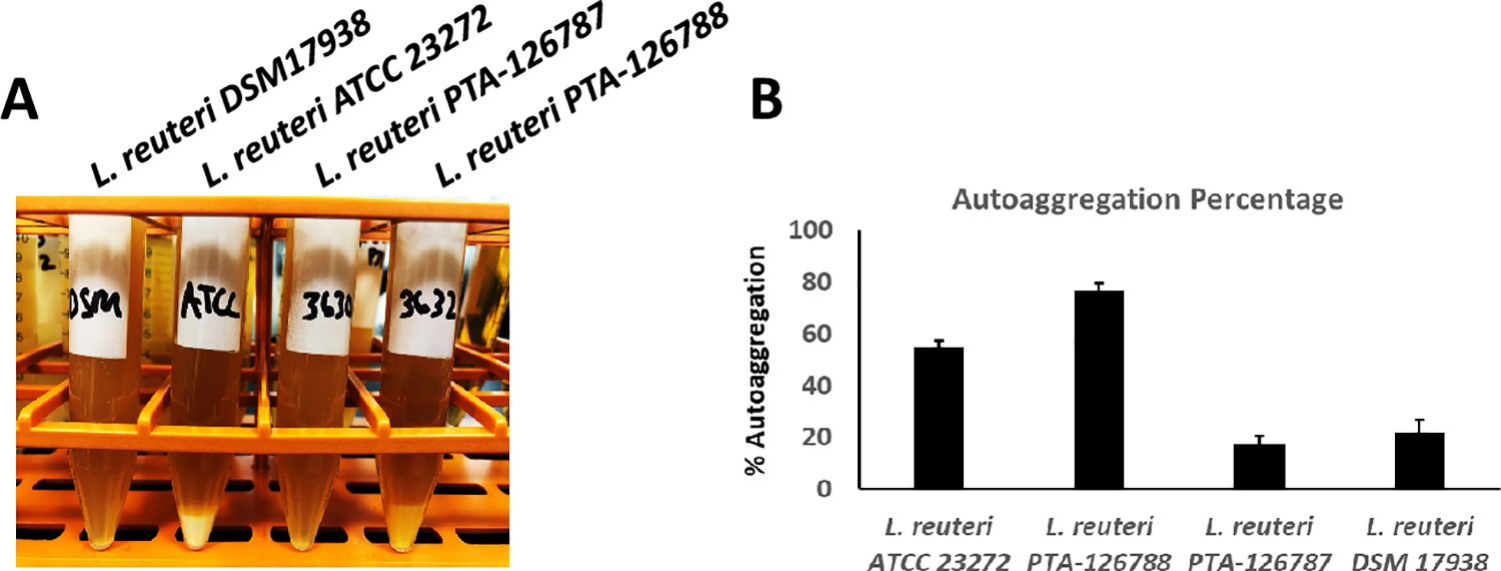
Ability of *L*. *reuteri* strains to undergo autoaggregation. **A**. Ability to undergo autoaggregation was determined by growing the strains overnight in MRS broth and observing for aggregate formation. **B**. Autoaggregation was quantified by measuring the OD_600_ in PBS after incubation for 5 hours and calculating the autoaggregation % as described in the methods section. The data represents the mean ± SE of 3 independent experiments.

Autoaggregation is a phenomenon where bacteria form fibrous-like aggregates after overnight growth and settle to the bottom of the tube. Once the bacteria are aggregated, they generally do not redisperse unless vigorously mixed manually. Autoaggregation appears to be one of the key properties needed for probiotic strains to attach to the epithelial cells in the gastrointestinal tract. The ability to aggregate has also been suggested to play a role in preventing pathogen colonization. As shown in **Fig 7A**, *L*. *reuteri* PTA-126788 showed excellent ability to autoaggregate, while the other *L*. *reuteri* strains PTA-126787 and DSM 17938 showed no ability to form aggregates. Similar to *L*. *reuteri* PTA-126788, the positive control, *L*. *reuteri* ATCC 23272 also showed ability to autoaggregate (**Fig 7A**). Quantification of autoaggregation showed that *L*. *reuteri* PTA-126788 exhibited the highest ability to autoaggregate, while *L*. *reuteri* strains PTA-126787 and DSM 17938 had the least ability to form aggregates (**Fig 7B**). The positive control *L*. *reuteri* ATCC 23272 showed moderate ability to autoaggregate (**Fig 7B**).

### Hydrogen peroxide production

**Fig 8:**
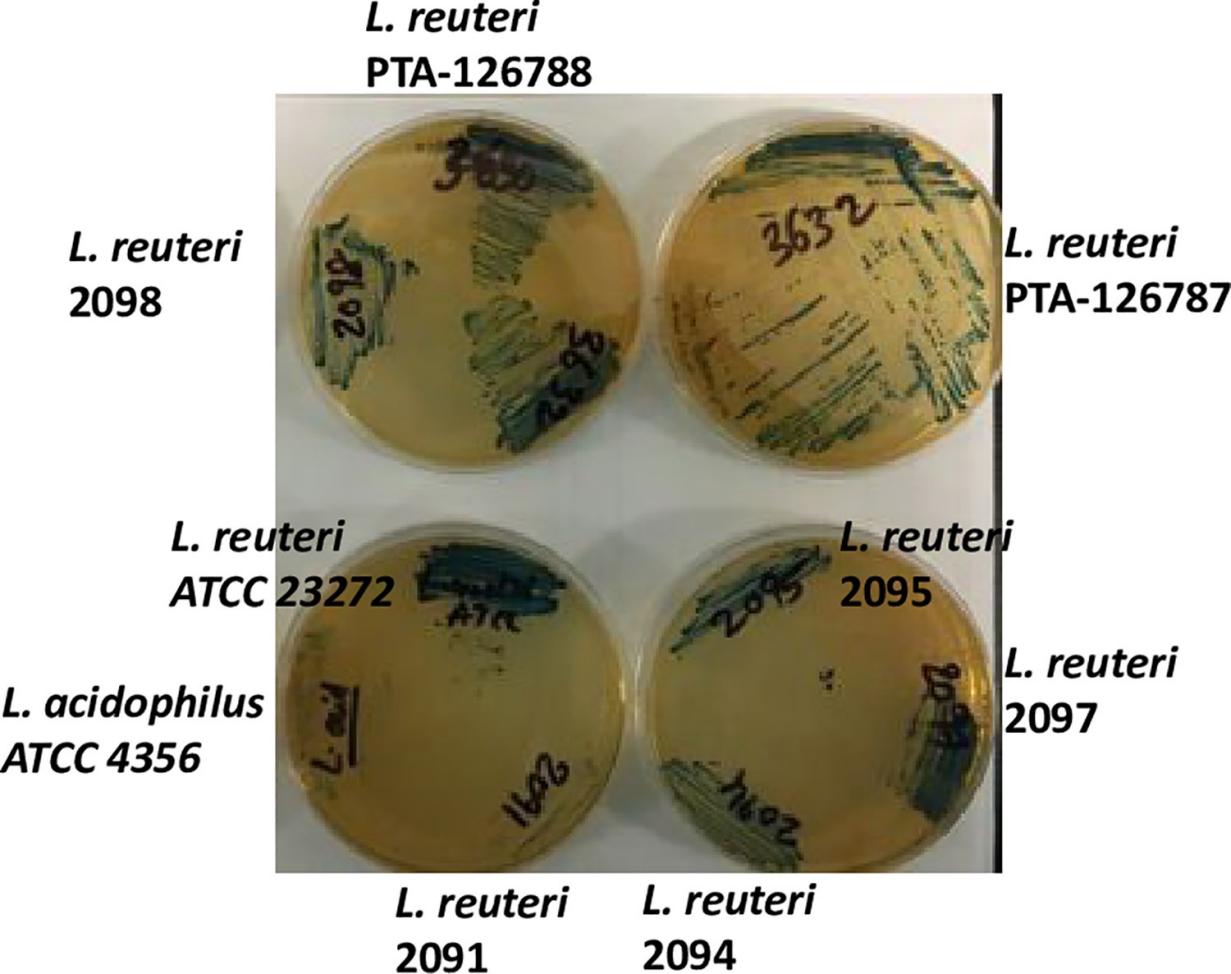
Ability of *L*. *reuteri* strains to produce hydrogen peroxide. Hydrogen peroxide production was assessed by growing the strains on MRS agar supplemented with 0.25mg/ml of tetramethylbenzidine and 0.01mg/ml of horseradish peroxidase and observing for color change. Dark blue coloration indicates high production of hydrogen peroxide. The data are representative of 3 independent experiments.

The ability of probiotic strains to produce hydrogen peroxide at physiological levels is highly desirable. Hydrogen peroxide production by *Lactobacillus johnsonii* NCC533 has been attributed to inducing recovery of the epithelial barrier and remission in inflammatory bowel disease [60]. Similarly, an *L*. *reuteri* probiotic strain ATCC PTA 5289 producing hydrogen peroxide was able to significantly reduce proinflammatory response and improved clinical outcomes in human patients with chronic periodontitis [61]. All the *L*. *reuteri* strains including PTA-126787 and PTA-126788 strains showed moderate to high ability to produce hydrogen peroxide as shown in **Fig 8**.

### Resistance to bile

**Fig 9:**
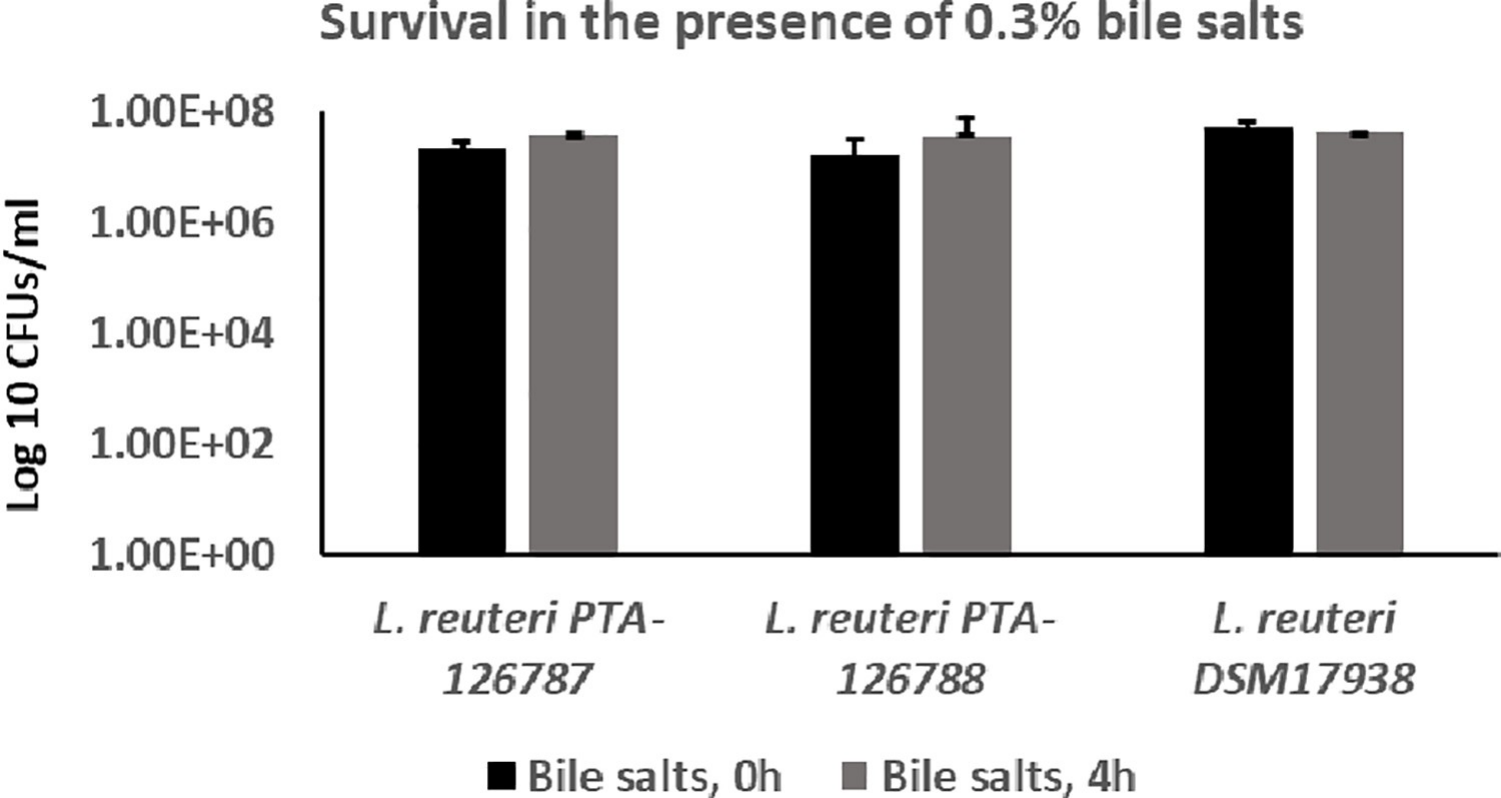
Tolerance of *L*. *reuteri* strains to 0.3% bile. The ability of *L*. *reuteri* strains to tolerate bile salts was assessed by growing the strains in the presence of 0.3% bile salts for 4 hours and determining the CFU counts at 0 hours and 4 hours after incubation with bile salts. The data represent the mean ± SD from 3 independent experiments.

The ability of the two *L*. *reuteri* strains to tolerate bile salts was also assessed by incubating the strains in the presence of 0.3% bile salts. The viability of *L*. *reuteri* PTA-126787, PTA-126788 and DSM 17938 did not change after incubation with 0.3% bile salts for 4 hours, suggesting that our strains are resistant to 0.3% bile salts similar to DSM 17938 (**Fig 9**).

### Resistance to acidic pH

**Fig 10:**
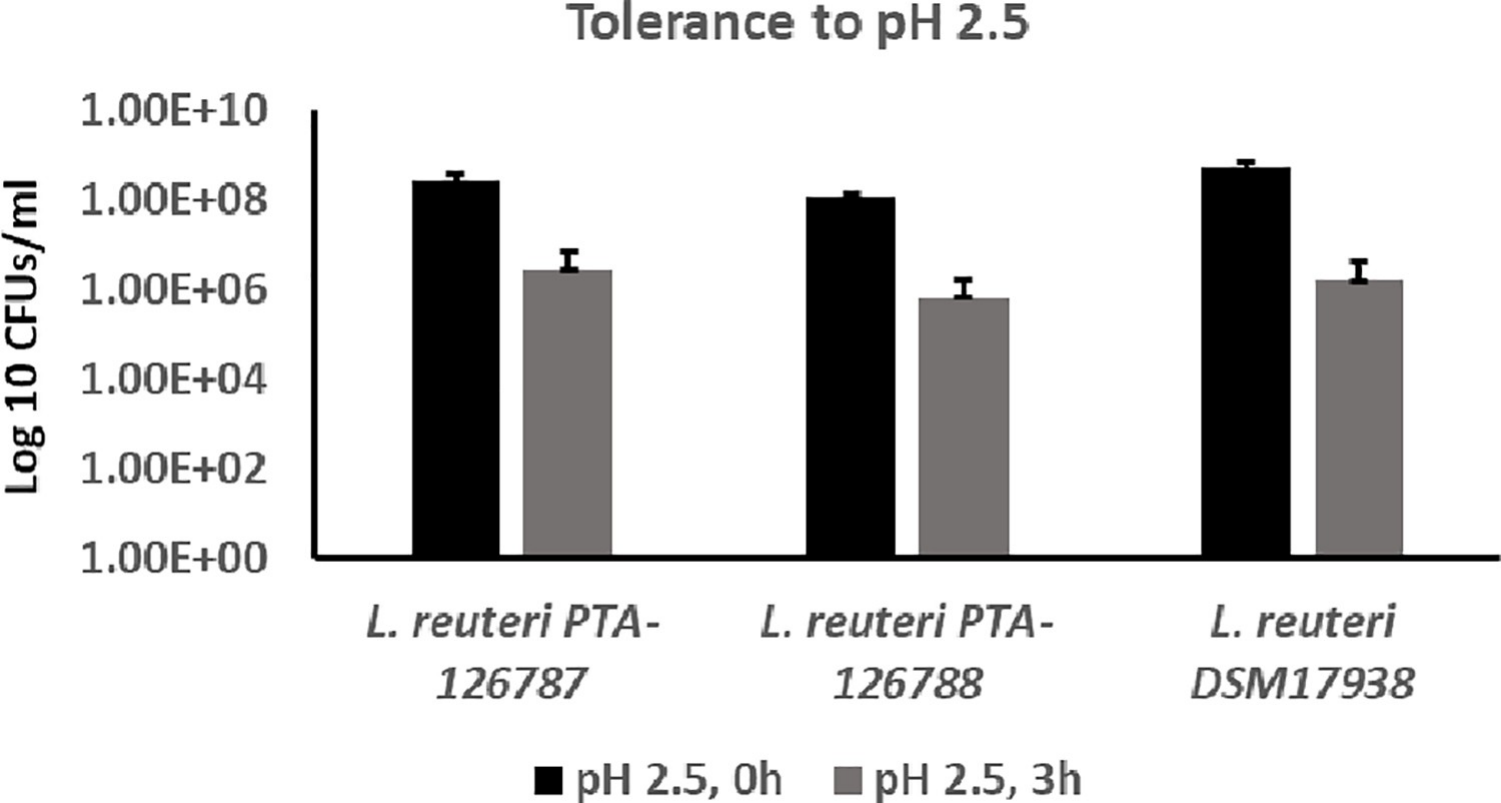
Tolerance of *L*. *reuteri* strains to acidic pH. The ability of *L*. *reuteri* strains to tolerate acidic pH was assessed by growing the strains at pH 2.5 for 3 hours and determining the CFU counts at 0 hours and 3 hours after incubation. The data represent the mean ± SD from 3 independent experiments.

The viability of *L*. *reuteri* PTA-126787 decreased from 2.81 x 10^8^ to 2.75 x 10^6^ after incubation at pH 2.5 for 3 hours (**Fig 10**). Similarly, the viability of *L*. *reuteri* PTA-126788 decreased from 1.22 x 10^8^ to 6.67 x 10^5^ after 3 hours incubation at pH 2.5 (**Fig 10**). Overall, there was approximately 2-log reduction in viability for PTA-126787 and approximately 2.5-log reduction in viability for PTA-126788 after incubation at pH 2.5 for 3 hours (**Fig 10**). The control strain DSM 17938 also showed approximately 2.5-log reduction in CFU counts from 5.51 x 10^8^ to 1.57 x 10^6^ after 3 hours incubation at pH 2.5 (**Fig 10**).

### *In vivo* safety in broilers

Compared to untreated control, members of groups treated with *L*. *reuteri* PTA-126787 and PTA-126788 daily in drinking water had no mortality, morbidity or adverse events in broiler chickens. Necropsy on day 26 showed no gross lesions indicative of health issues. Compared to the control group, histopathological analysis of trachea, lung and cecal tonsils from the groups treated with *L*. *reuteri* PTA-126787 or PTA-126788 showed no evidence of inflammation or abnormal pathology compared to untreated group (**S2 Fig**).

## Discussion

Our understanding of the microbiome and its impact on human and animal health is rapidly evolving, leading to the identification of innovative ways to impact human and animal diseases. Undoubtedly, the past two decades have witnessed a tremendous progress in the area of probiotics highlighting their key role in supporting general health, enhancing immune function and showing the potential to reduce specific diseases. Research has repeatedly shown that the survival, safety, and efficacy properties of probiotic candidates are strain specific and cannot be generalized. In the present study, we isolated two novel *Lactobacillus* strains from chicken cecum, identified them as *L*. *reuteri* and established their safety using various genomic, *in vitro*, and *in vivo* studies, supporting their application as potential probiotics for human and animal health.

Identification is the first step in establishing the safety of a probiotic candidate and regulatory agencies recommend that at least two state-of-the-art methods be used to correctly identify a probiotic candidate [35, 62]. API 50 CHL analysis identified our strains as *L*. *fermentum*. *L*. *reuteri* is a subtype of *L*. *fermentum* and the two species are indistinguishable at the biochemical level [63]. 16S rRNA identification confirmed that our strains have closest homology to *L*. *reuteri* strains. Whole-genome sequencing coupled with phylogenetic analyses further confirmed that our strains have closest relatedness to *L*. *reuteri* and that our strains genetically cluster with DSM 17938, SD2112 (parent strain of DSM 17938) and RC-14. DSM 17938 and RC-14 are widely used as part of several commercially marketed dietary supplements and functional foods and there exists a plethora of clinical evidence supporting their safety and efficacy for different disease indications in humans. Consistent with our findings, several previous whole-genome phylogenetic studies also showed that the parent strain of DSM 17938, SD2112 indeed clusters with poultry isolates under poultry/human lineage IV and the authors from these studies hypothesized that SD2112 may have indeed originated from poultry [64–67]. All together, these findings clearly establish that our strains belong to *L*. *reuteri* species and that our strains have closest homology to the two commercially marketed probiotic candidates with proven human clinical safety, DSM 17938 and RC-14.

Long read sequencing technology enabled complete genome characterization with each chromosome and plasmid represented by large, nearly complete contigs. Comprehensive functional annotation of the *L*. *reuteri* strains PTA-126787 and PTA-126788 revealed presence of several genes important for probiotic efficacy. Probiotic bacteria are known to contain bioactive secondary metabolites that interact with other pathogenic bacteria to attenuate virulence [68–71]. For instance, lactic acids produced by lactic acid bacteria inhibit the growth and survival of nearby pathogens and inactivate human immunodeficiency virus by increasing acidity of the surrounding environment [72]. Both PTA-126787 and PTA-126788 strains contain four coding sequences encoding D-lactate dehydrogenase (EC 1.1.1.28) and four encoding L-lactate dehydrogenase (EC 1.1.1.27) which are responsible for lactic acid production. However, IVR12_00498 from strain PTA-126787 is a pseudogene due to a frameshift mutation.

One of the key desirable traits in a probiotic candidate is the ability to adhere to epithelial cells. The genes identified in both strains of *L*. *reuteri* putatively encode proteins involved in adhesion, providing stability to the strains and the ability to compete with other undesirable resident gut bacteria, thereby enabling effective colonization of the gut and exclusion of pathogens [73, 74]. Sortase-dependent proteins are an important group of cell surface proteins in *Lactobacillus spp*. and are responsible for sorting various kinds of cell surface proteins, thus playing an important role in adhesion [75]. The genomes of both strains contain the gene encoding phosphate starvation-inducible protein PhoH, a member of both the Pho regulon and the sB-regulated general stress regulon. Pho regulon plays a key role in regulating phosphate homeostasis and is generally induced in response to phosphate starvation. The sB-dependent general stress proteins are predicted to provide cells with several kinds of non-specific stress tolerance [76].

While the diversity of phages in gut ecosystems is getting increasingly well-characterized, knowledge is limited on how phages contribute to the evolution and ecology of their host bacteria [77, 78]. Prophage analysis of *L*. *reuteri* strain DSM 17938 showed 5 prophage regions while the strains, PTA-126787 and PTA-126788 had 6 and 8 prophage regions, respectively (**S9 Table**). Prophages can be advantageous for gut symbionts like *L*. *reuteri* by increasing its competitiveness in the intestinal niche [77].

Genome analysis identified the presence of *tetW* in both *L*. *reuteri* strains. *tetW* was found to be present on the chromosome and no elements indicative of horizontal transfer (plasmids, phages, transposons, or conjugation elements) were identified in the 15-kb flanking regions on both sides of *tetW* (**S2 File**). Phenotypic analysis showed that the two strains are susceptible to all clinically relevant antimicrobials with MICs below the EFSA recommended microbiological cut offs, except for tetracycline. For tetracycline, both strains showed a marginal 2-fold increase in MIC than the recommended microbiological cut off and a 2-fold variation in the MIC is considered acceptable due to technical variation in the MIC assay and hence the strains can be considered phenotypically susceptible [59, 79]. Together, these data suggest that the presence of *tetW* in our *L*. *reuteri* poses minimal risk to human and animal health.

During carbohydrate fermentation, *Lactobacillus* species are known to produce either exclusively L-lactic acid, exclusively D-lactic acid or a racemic mix of L- and D-lactic acid [80]. Many commercially used *Lactobacillus* species produce a racemic mix of L- and D-lactic acid, including the most widely used *L*. *reuteri* probiotic strains DSM 17938 and NCIMB 30242 [81–84]. Screening for D-lactic acid has gained much attention due to D-lactic acidosis and encephalopathy reported in individuals with short bowel syndrome and intestinal failure [85–87]. However, such illnesses have not yet been reported in healthy individuals. Quantification of L- and D-lactic acid showed that our strains produce a racemic mix of L- and D-lactic acid with predominance of L-lactic acid. Consistent with the previous reports, DSM 17938 also produced a racemic mix of D- and L-lactic acid [81]. NCIMB 30242, another widely used *L*. *reuteri* probiotic strain, also produces a racemic mix of D- and L-lactic acid in a ratio of 9:11 [55]. Many clinical studies conducted on DSM 17938 and NCIMB 30242 in infants, children and adults showed no evidence of adverse effects from D-lactic acidosis [26].

*Lactobacillus* species also possess amino acid decarboxylase activity, which results in production of toxic metabolites such as histamine, tyramine, cadaverine and putrescine. Toxicity from biogenic amines are rare but when reported is mostly associated with histamine and less commonly with tyramine [88–90]. Genome analysis showed that our strains do not encode for any known genes encoding for histamine or tyramine production. Analysis of the strains for their ability to produce biogenic amines using decarboxylase media developed by Bover-Cid and Holzapfei [56] showed that our strains are not capable of producing histamine or tyramine. The data clearly suggest that our strains do not produce the two major biogenic amines associated with toxicity in humans—histamine, and tyramine.

Our bioinformatic search identified a CDS predicted to encode arginine deiminase in both *L*. *reuteri* PTA-126787 and PTA-126788. Arginine deiminase is a common enzyme present in most lactic acid bacteria and is used to convert arginine into ornithine via citrulline and allows bacteria to adapt to non-optimal stress conditions such as acid, osmotic and temperature stresses [91]. Expectedly, a gene encoding arginine deiminase was also present in the genome of the commercially marketed *L*. *reuteri* strain DSM 17938 (Accession no. WP_003670382.1). Our bioinformatics analysis showed that the downstream gene ornithine decarboxylase required for putrescine production is absent in the genomes of *L*. *reuteri* PTA-126787 and PTA-126788. Consistent with this, *in vitro* analysis of biogenic amines using decarboxylase media showed that our strains are not capable of producing putrescine using L-ornithine as a substrate. Thus, the presence of arginine deiminase may not result in production of the harmful biogenic amine putrescine.

Studies on the survival properties of probiotic candidates in simulated gastrointestinal conditions are key to our understanding of their safety and efficacy. Probiotic candidates are exposed to a variety of harsh extremes in the gastrointestinal tract, but acidic pH of the stomach and bile salts appear to be the dominant factors determining the survival and growth of probiotic candidates in the gastrointestinal tract. The chicken duodenum has a typical bile salt concentration of 0.175% and likewise, the human duodenum has a bile salt concentration of around 0.3% [92, 93]. Both of our *L*. *reuteri* strains showed a similar survival profile to that of commercially marketed probiotic *L*. *reuteri* DSM 17938 in the presence of 0.3% bile salts. The human and chicken (proventriculus) stomachs have a pH of around 1.5–4.0 [94, 95]. Both of our strains showed similar survival at pH 2.5, similar to DSM 17938. Together, these data suggest that the two probiotic candidates possess desirable survival properties in a simulated gastrointestinal environment.

In the present study, broiler chickens (SPF White Leghorn chickens) were used as a model for preliminary screening of *L*. *reuteri* PTA-126787 and PTA-126788 strains for gross safety parameters. Our data showed that daily administration of the two *L*. *reuteri* strains for 26 days to chickens was safe and did not induce any adverse events. The data provided here serves as a preliminary safety evidence of the two probiotic candidates for potential animal health applications. Future studies will focus on further safety evaluation of the two strains in a rat toxicity model for potential human health applications.

In conclusion, we provide comprehensive genomic, *in vitro*, and *in vivo* evidence to support the safety of two novel *L*. *reuteri* candidates, PTA-126787 and PTA-126788. These findings would serve as the basis for designing future studies to establish efficacy in humans as well as animals.

## Supporting information

S1 Fig Genome mapping of the *L*. *reuteri* strains PTA-126787 and PTA-126788 showing regions and positions occupied by different types of prophages as predicted by PhiSpy. (TIF)

S2 Fig Mean histopathological scores and representative histopathology pictures of cecal tonsils, lungs, and trachea. (TIF)

S1 Table Orthologs shared between *L*. *reuteri* strains. (DOCX)

S2 Table Summary of ortholog statistics of *L*. *reuteri* strains. (DOCX)

S3 Table Unique genes in *L*. *reuteri* strains PTA-126787 and PTA-126788. (DOCX)

S4 Table IS elements identified in *L*. *reuteri* PTA-126787 and PTA-126788 genomes. (DOCX)

S5 Table Predicted antimicrobial resistance genes in *L*. *reuteri* strains PTA-126787 and PTA-126788. (DOCX)

S6 Table Identified protein-coding genes putative for arginine deiminase pathway. (DOCX)

S7 Table Putative genes in PTA-126787 and PTA-126788 involved in lactic acids production. (DOCX)

S8 Table Identified protein-encoding genes putative for adhesion by *L*. *reuteri* strains PTA-126787 and PTA-126788. (DOCX)

S9 Table Prophage regions in *L*. *reuteri* strains DSM 17938, PTA-126787 and PTA-126788. (DOCX)

S1 File tetW along with flanking regions with no homology to known mobile genetic elements. (XLSX)

S2 File (PDF)

S1 Raw images (PDF)
