# Supplementary figures and images for: In silico, in vitro and in vivo safety evaluation of Limosilactobacillus reuteri strains ATCC PTA-126787 & ATCC PTA-126788 for potential probiotic applications
Source: PLoS One. 2022 Jan 26;17(1):e0262663. doi: 10.1371/journal.pone.0262663 (PMC8791467; doi:10.1371/journal.pone.0262663)

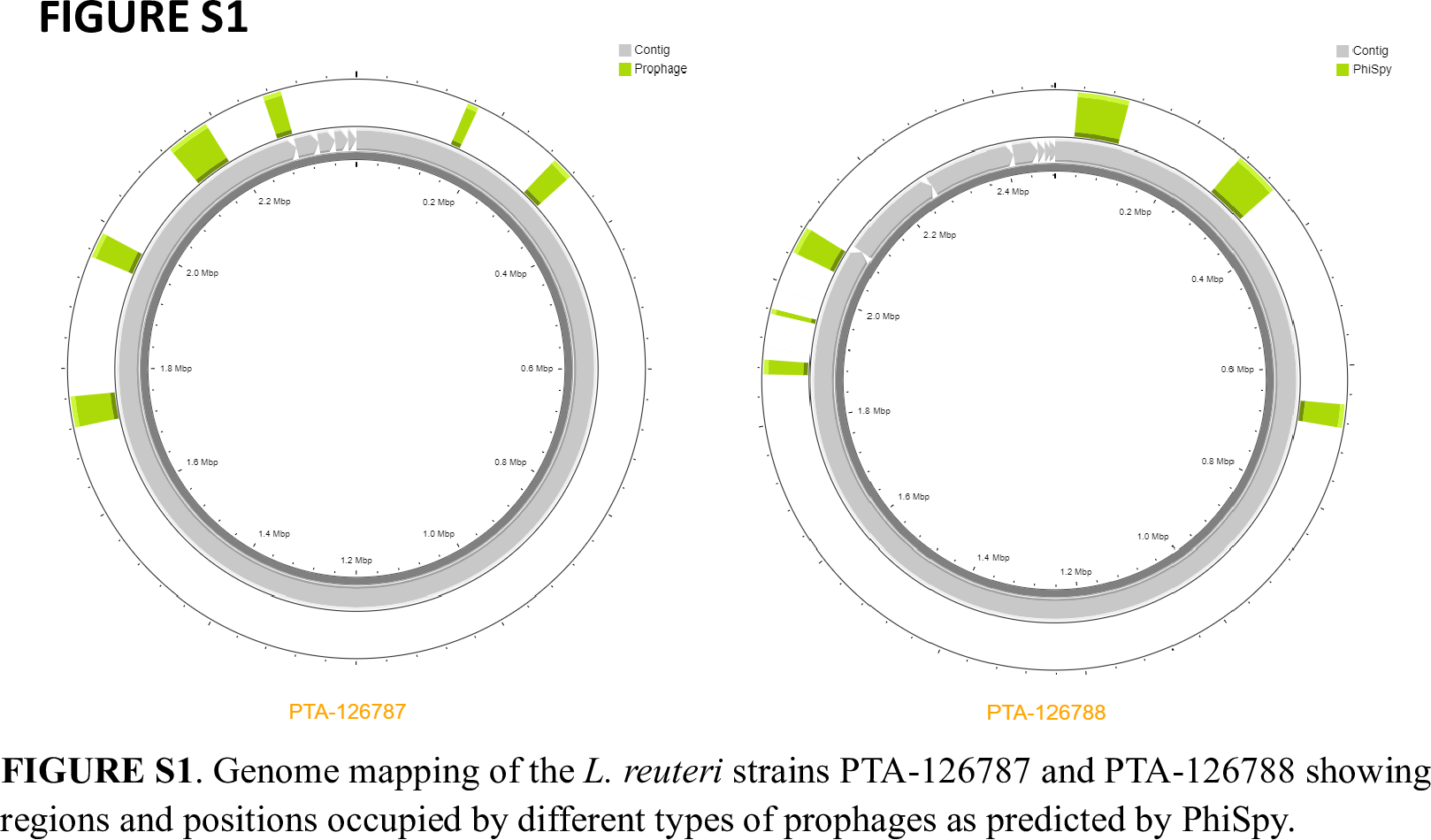

Supplement: S1 Fig — (TIF) [file pone.0262663.s001.tif]

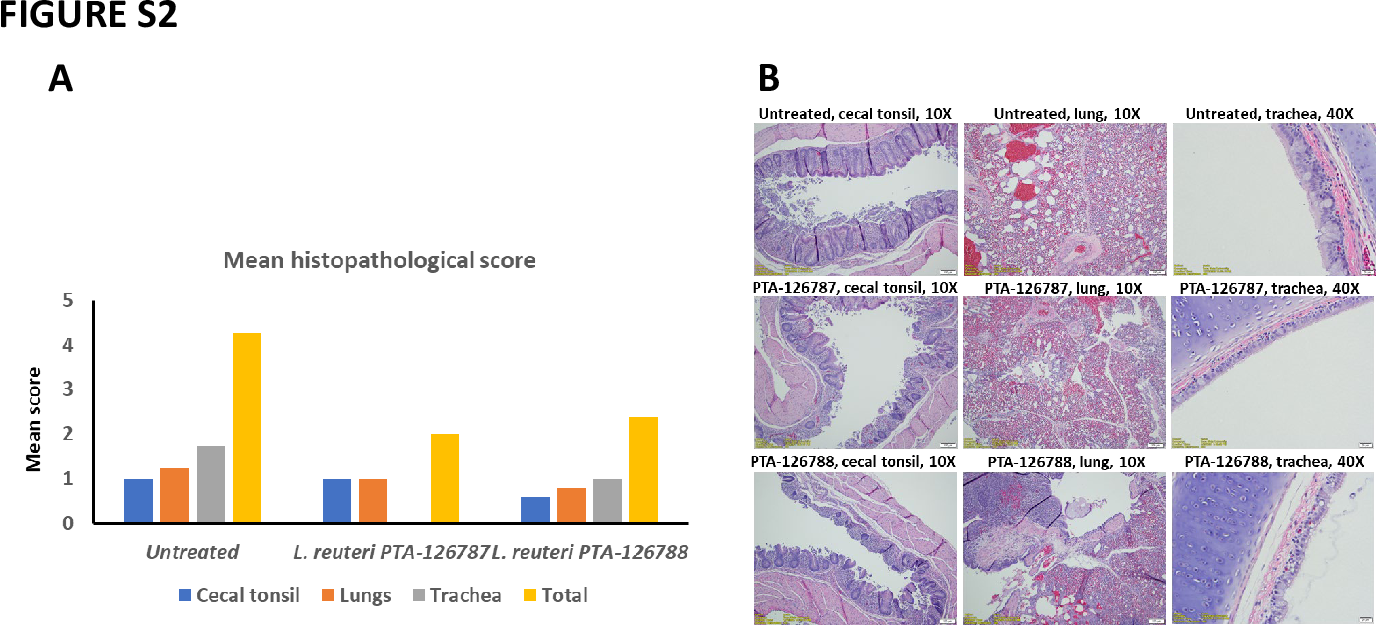

Supplement: S2 Fig — (TIF) [file pone.0262663.s002.tif]

Agarose 1%

M 1 2 3 4 5 6 7 8 9 10

Figure 1A

X X X 1 2 3 4 5 6 7 M

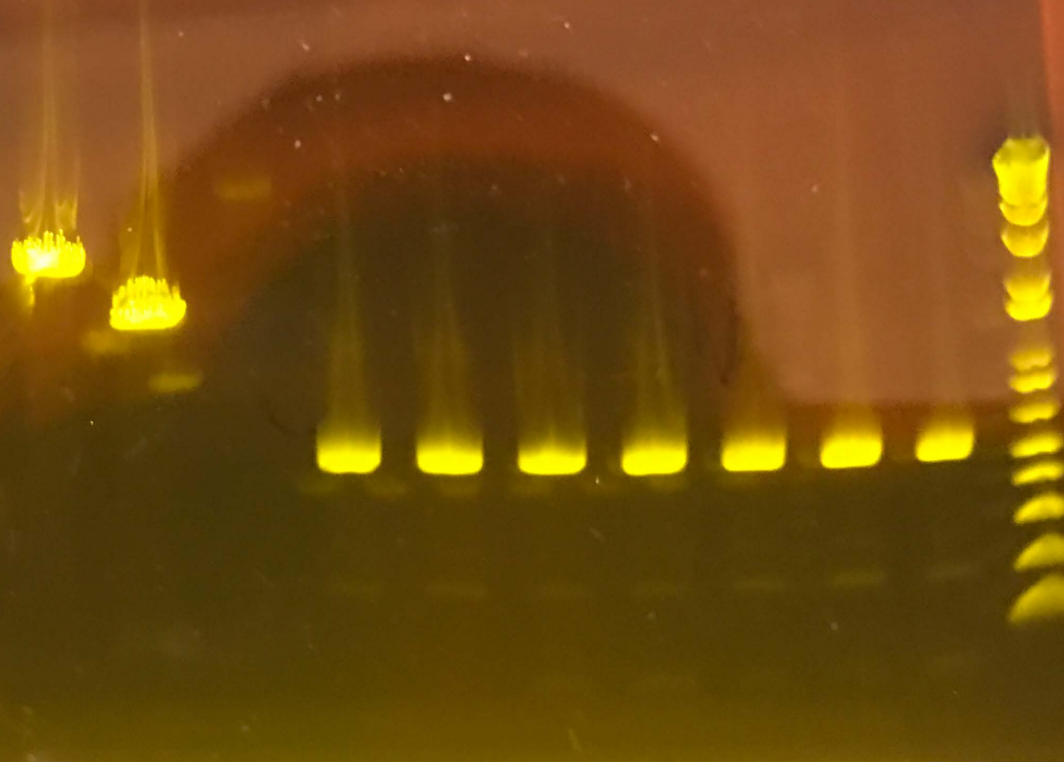

E-Gel® EX  
invitrogen

Supplement: S1 Raw images — (PDF) [file pone.0262663.s014.pdf]
