# Supplementary material for: In silico, in vitro and in vivo safety evaluation of Limosilactobacillus reuteri strains ATCC PTA-126787 & ATCC PTA-126788 for potential probiotic applications
Source: PLoS One. 2022 Jan 26;17(1):e0262663. doi: 10.1371/journal.pone.0262663 (PMC8791467; doi:10.1371/journal.pone.0262663)
Supplement: S1 Table — (DOCX) [file pone.0262663.s003.docx]

**S1 Table.** Orthologs shared between L. reuteri strains.

|  | **ATCC53608** | **CF48-3A** | **DSM20016** | **PTA-126787** | **PTA-126788** | **SD2112** |
| --- | --- | --- | --- | --- | --- | --- |
| ATCC53608 | 0 | 1597 | 1612 | 1572 | 1523 | 1612 |
| CF48-3A | 1568 | 0 | 1610 | 1680 | 1631 | 2082 |
| DSM20016 | 1583 | 1609 | 0 | 1621 | 1569 | 1614 |
| PTA-126787 | 1582 | 1696 | 1628 | 0 | 2264 | 1715 |
| PTA-126788 | 1553 | 1659 | 1589 | 2242 | 0 | 1677 |
| SD2112 | 1660 | 2148 | 1661 | 1788 | 1745 | 0 |
