# Supplementary material for: In silico, in vitro and in vivo safety evaluation of Limosilactobacillus reuteri strains ATCC PTA-126787 & ATCC PTA-126788 for potential probiotic applications
Source: PLoS One. 2022 Jan 26;17(1):e0262663. doi: 10.1371/journal.pone.0262663 (PMC8791467; doi:10.1371/journal.pone.0262663)
Supplement: S2 Table — (DOCX) [file pone.0262663.s004.docx]

**S2 Table.** Summary of ortholog statistics of L. reuteri strains.

|  | **ATCC53608** | **CF48-3A** | **DSM20016** | **PTA-126787** | **PTA-126788** | **SD2112** |
| --- | --- | --- | --- | --- | --- | --- |
| Number of genes | 1906 | 2164 | 1860 | 2397 | 2416 | 2300 |
| Number of genes in orthogroups | 1764 | 2119 | 1769 | 2387 | 2303 | 2292 |
| Number of unassigned genes | 142 | 45 | 91 | 10 | 113 | 8 |
| Percentage of genes in orthogroups | 92.5 | 97.9 | 95.1 | 99.6 | 95.3 | 99.7 |
| Percentage of unassigned genes | 7.5 | 2.1 | 4.9 | 0.4 | 4.7 | 0.3 |
| Number of orthogroups containing species | 1652 | 2017 | 1706 | 2177 | 2123 | 2008 |
| Percentage of orthogroups containing species | 63.8 | 77.8 | 65.8 | 84 | 81.9 | 77.5 |
| Number of species-specific orthogroups | 5 | 8 | 0 | 13 | 7 | 0 |
| Number of genes in species-specific orthogroups | 10 | 16 | 0 | 26 | 15 | 0 |
| Percentage of genes in species-specific orthogroups | 0.5 | 0.7 | 0 | 1.1 | 0.6 | 0 |
