# Supplementary material for: In silico, in vitro and in vivo safety evaluation of Limosilactobacillus reuteri strains ATCC PTA-126787 & ATCC PTA-126788 for potential probiotic applications
Source: PLoS One. 2022 Jan 26;17(1):e0262663. doi: 10.1371/journal.pone.0262663 (PMC8791467; doi:10.1371/journal.pone.0262663)
Supplement: S3 Table — (DOCX) [file pone.0262663.s005.docx]

**S3 Table**. Genes unique to *L. reuteri* PTA-126787 and PTA-126788.

| Orthogroup | | PTA-126787 | PTA-126788 | Annotation |
| --- | --- | --- | --- | --- |
| OG0002869 | IU404_00186 | |  | hypothetical protein |
| OG0002870 | IU404_00494 | |  | Phosphate-binding protein PstS 1 |
| OG0002871 | IU404_00793 | |  | hypothetical protein |
| OG0002872 | IU404_01929 | |  | 30S ribosomal protein S1 |
| OG0002873 | IU404_02246 | |  | hypothetical protein |
| OG0002874 | IU404_02369 | |  | 50S ribosomal protein L19 |
| OG0002875 | IU404_02438 | |  | hypothetical protein |
| OG0002876 | IU404_02439 | |  | hypothetical protein |
| OG0002877 | IU404_02440 | |  | hypothetical protein |
| OG0002878 | IU404_02495 | |  | hypothetical protein |
| OG0002959 |  | | IVR12_02318 | hypothetical protein |
| OG0002879 |  | | IVR12_00335 | hypothetical protein |
| OG0002880 |  | | IVR12_00390 | hypothetical protein |
| OG0002881 |  | | IVR12_00406 | hypothetical protein |
| OG0002882 |  | | IVR12_00450 | Argininosuccinate synthase |
| OG0002883 |  | | IVR12_00667 | hypothetical protein |
| OG0002884 |  | | IVR12_00951 | hypothetical protein |
| OG0002885 |  | | IVR12_01333 | UDP-N-acetylmuramoyl-L-alanyl-D-glutamate--L- lysine ligase |
| OG0002886 |  | | IVR12_01986 | hypothetical protein |
| OG0002887 |  | | IVR12_02184 | hypothetical protein |
| OG0002888 |  | | IVR12_02193 | hypothetical protein |
| OG0002889 |  | | IVR12_02204 | hypothetical protein |
| OG0002890 |  | | IVR12_02210 | UDP-N-acetylmuramate--L-alanine ligase |
| OG0002891 |  | | IVR12_02211 | Inner membrane protein YbhL |
| OG0002892 |  | | IVR12_02212 | hypothetical protein |
| OG0002893 |  | | IVR12_02213 | DNA polymerase I |
| OG0002894 |  | | IVR12_02214 | Formamidopyrimidine-DNA glycosylase |
| OG0002895 |  | | IVR12_02215 | Dephospho-CoA kinase |
| OG0002896 |  | | IVR12_02217 | Replication initiation and membrane attachment protein |
| OG0002897 |  | | IVR12_02218 | Primosomal protein DnaI |
| OG0002898 |  | | IVR12_02219 | hypothetical protein |
| OG0002899 |  | | IVR12_02220 | Translation initiation factor IF-3 |
| OG0002900 |  | | IVR12_02221 | 50S ribosomal protein L35 |
| OG0002901 |  | | IVR12_02222 | 50S ribosomal protein L20 |
| OG0002902 |  | | IVR12_02223 | hypothetical protein |
| OG0002903 |  | | IVR12_02224 | hypothetical protein |
| OG0002904 |  | | IVR12_02225 | hypothetical protein |
| OG0002905 |  | | IVR12_02228 | hypothetical protein |
| OG0002906 |  | | IVR12_02230 | putative protein YqeH |
| OG0002907 |  | | IVR12_02231 | RNA-binding protein |
| OG0002908 |  | | IVR12_02234 | Ribosomal silencing factor RsfS |
| OG0002909 |  | | IVR12_02235 | 2-methoxy-6-polyprenyl-1,4-benzoquinol methylase, mitochondrial |
| OG0002910 |  | | IVR12_02237 | hypothetical protein |
| OG0002911 |  | | IVR12_02238 | 50S ribosomal protein L32 |
| OG0002912 |  | | IVR12_02240 | Adaptive-response sensory-kinase SasA |
| OG0002913 |  | | IVR12_02242 | Inner membrane transporter YjeM |
| OG0002914 |  | | IVR12_02243 | Multidrug export protein MepA |
| OG0002915 |  | | IVR12_02245 | Membrane protein insertase MisCB |
| OG0002916 |  | | IVR12_02248 | 23S rRNA (guanosine-2'-O-)-methyltransferase RlmB |
| OG0002917 |  | | IVR12_02250 | HTH-type transcriptional regulator YodB |
| OG0002918 |  | | IVR12_02255 | Transcription elongation factor GreA |
| OG0002919 |  | | IVR12_02256 | hypothetical protein |
| OG0002920 |  | | IVR12_02258 | Penicillin-binding protein 2B |
| OG0002921 |  | | IVR12_02259 | Penicillin-binding protein 2B |
| OG0002922 |  | | IVR12_02260 | 50S ribosomal protein L33 1 |
| OG0002923 |  | | IVR12_02261 | putative protein YqgN |
| OG0002924 |  | | IVR12_02265 | Glucokinase |
| OG0002925 |  | | IVR12_02266 | putative protein YibN |
| OG0002926 |  | | IVR12_02267 | hypothetical protein |
| OG0002927 |  | | IVR12_02270 | Glutamate--tRNA ligase |
| OG0002928 |  | | IVR12_02271 | putative N-acetyl-LL-diaminopimelate aminotransferase |
| OG0002929 |  | | IVR12_02272 | IS30 family transposase ISHahy10 |
| OG0002930 |  | | IVR12_02273 | Aspartate/prephenate aminotransferase |
| OG0002931 |  | | IVR12_02274 | Aspartate aminotransferase |
| OG0002932 |  | | IVR12_02275 | putative N-acetyl-LL-diaminopimelate aminotransferase |
| OG0002933 |  | | IVR12_02276 | hypothetical protein |
| OG0002934 |  | | IVR12_02280 | Tyrosine recombinase XerC |
| OG0002935 |  | | IVR12_02282 | hypothetical protein |
| OG0002936 |  | | IVR12_02283 | hypothetical protein |
| OG0002937 |  | | IVR12_02284 | hypothetical protein |
| OG0002938 |  | | IVR12_02285 | hypothetical protein |
| OG0002939 |  | | IVR12_02286 | hypothetical protein |
| OG0002940 |  | | IVR12_02287 | hypothetical protein |
| OG0002941 |  | | IVR12_02288 | hypothetical protein |
| OG0002942 |  | | IVR12_02289 | hypothetical protein |
| OG0002943 |  | | IVR12_02290 | hypothetical protein |
| OG0002944 |  | | IVR12_02291 | hypothetical protein |
| OG0002945 |  | | IVR12_02294 | hypothetical protein |
| OG0002946 |  | | IVR12_02295 | hypothetical protein |
| OG0002947 |  | | IVR12_02296 | hypothetical protein |
| OG0002948 |  | | IVR12_02299 | hypothetical protein |
| OG0002949 |  | | IVR12_02300 | hypothetical protein |
| OG0002950 |  | | IVR12_02305 | hypothetical protein |
| OG0002951 |  | | IVR12_02307 | hypothetical protein |
| OG0002952 |  | | IVR12_02308 | hypothetical protein |
| OG0002953 |  | | IVR12_02310 | hypothetical protein |
| OG0002954 |  | | IVR12_02312 | hypothetical protein |
| OG0002955 |  | | IVR12_02313 | hypothetical protein |
| OG0002956 |  | | IVR12_02314 | hypothetical protein |
| OG0002957 |  | | IVR12_02316 | Endoribonuclease PemK |
| OG0002958 |  | | IVR12_02317 | Antitoxin MazE |
| OG0002959 |  | | IVR12_02318 | hypothetical protein |
| OG0002960 |  | | IVR12_02319 | hypothetical protein |
| OG0002961 |  | | IVR12_02320 | hypothetical protein |
| OG0002962 |  | | IVR12_02321 | hypothetical protein |
| OG0002963 |  | | IVR12_02322 | hypothetical protein |
| OG0002964 |  | | IVR12_02323 | hypothetical protein |
| OG0002965 |  | | IVR12_02324 | Chromosome partition protein Smc |
| OG0002966 |  | | IVR12_02325 | hypothetical protein |
| OG0002967 |  | | IVR12_02326 | hypothetical protein |
| OG0002968 |  | | IVR12_02327 | hypothetical protein |
| OG0002969 |  | | IVR12_02329 | Tyrosine recombinase XerC |
| OG0002970 |  | | IVR12_02330 | hypothetical protein |
| OG0002971 |  | | IVR12_02331 | hypothetical protein |
| OG0002972 |  | | IVR12_02333 | hypothetical protein |
| OG0002973 |  | | IVR12_02334 | hypothetical protein |
| OG0002974 |  | | IVR12_02335 | hypothetical protein |
| OG0002975 |  | | IVR12_02336 | hypothetical protein |
| OG0002976 |  | | IVR12_02337 | hypothetical protein |
| OG0002977 |  | | IVR12_02338 | Glutaredoxin-like protein NrdH |
| OG0002978 |  | | IVR12_02339 | hypothetical protein |
| OG0002979 |  | | IVR12_02340 | DNA topoisomerase 3 |
| OG0002980 |  | | IVR12_02346 | hypothetical protein |
| OG0002981 |  | | IVR12_02348 | hypothetical protein |
| OG0002982 |  | | IVR12_02350 | Signal peptidase IB |
| OG0002983 |  | | IVR12_02351 | hypothetical protein |
| OG0002984 |  | | IVR12_02352 | hypothetical protein |
| OG0002985 |  | | IVR12_02390 | hypothetical protein |
| OG0002986 |  | | IVR12_02440 | hypothetical protein |
| OG0002987 |  | | IVR12_02585 | hypothetical protein |
| OG0002988 |  | | IVR12_02601 | hypothetical protein |
| OG0002989 |  | | IVR12_02602 | hypothetical protein |
| OG0002990 |  | | IVR12_02603 | hypothetical protein |
| OG0002991 |  | | IVR12_02604 | hypothetical protein |
