# Supplementary material for: In silico, in vitro and in vivo safety evaluation of Limosilactobacillus reuteri strains ATCC PTA-126787 & ATCC PTA-126788 for potential probiotic applications
Source: PLoS One. 2022 Jan 26;17(1):e0262663. doi: 10.1371/journal.pone.0262663 (PMC8791467; doi:10.1371/journal.pone.0262663)
Supplement: S4 Table — (DOCX) [file pone.0262663.s006.docx]

**S4 Table**. IS elements identified in *L. reuteri* PTA-126787 and PTA-126788 genomes.

***L. reuteri* PTA-126787**

| **Sequence identifier** | **Family name of IS element** | **Number of IS copies** | **% of genome sequence content spanned by IS elements** | **Length of sequence segments spanned by IS elements in a sequence** | **Length of specific sequence** |
| --- | --- | --- | --- | --- | --- |
| IU404_1 | IS110 | 6 | 0.41 | 9431 | 2297299 |
| IU404_1 | IS1182 | 3 | 0.23 | 5390 | 2297299 |
| IU404_1 | IS200/IS605 | 19 | 1.35 | 31052 | 2297299 |
| IU404_1 | IS21 | 3 | 0.17 | 3921 | 2297299 |
| IU404_1 | IS3 | 5 | 0.35 | 7998 | 2297299 |
| IU404_1 | IS30 | 41 | 2.16 | 49546 | 2297299 |
| IU404_1 | IS66 | 5 | 0.42 | 9725 | 2297299 |
| IU404_1 | ISL3 | 1 | 0.04 | 806 | 2297299 |
| IU404_2 | IS30 | 2 | 44.04 | 16812 | 38177 |
| IU404_3 | IS30 | 1 | 4.56 | 1259 | 27631 |
| PTA-126787 | total | 86 | 5.67 | 135940 | 2399045 |

***L. reuteri* PTA-126788**

| **Sequence identifier** | **Family name of IS element** | **Number of IS copies** | **% genome sequence content spanned by IS elements** | **Length of sequence segments spanned by IS elements in a sequence** | **Length of specific sequence** |
| --- | --- | --- | --- | --- | --- |
| IVR12_1 | IS110 | 4 | 0.32 | 6649 | 2096256 |
| IVR12_1 | IS1182 | 2 | 0.17 | 3654 | 2096256 |
| IVR12_1 | IS200/IS605 | 16 | 1.21 | 25318 | 2096256 |
| IVR12_1 | IS21 | 3 | 0.21 | 4430 | 2096256 |
| IVR12_1 | IS3 | 5 | 0.38 | 7998 | 2096256 |
| IVR12_1 | IS30 | 36 | 2.16 | 45270 | 2096256 |
| IVR12_1 | IS66 | 5 | 0.46 | 9725 | 2096256 |
| IVR12_1 | ISL3 | 1 | 0.04 | 806 | 2096256 |
| IVR12_2 | IS200/IS605 | 4 | 3.76 | 6243 | 165920 |
| IVR12_2 | IS3 | 1 | 0.1 | 168 | 165920 |
| IVR12_2 | IS30 | 2 | 1.28 | 2118 | 165920 |
| IVR12_3 | IS110 | 2 | 1.98 | 2973 | 150023 |
| IVR12_3 | IS1182 | 1 | 1.19 | 1792 | 150023 |
| IVR12_3 | IS200/IS605 | 2 | 2.14 | 3214 | 150023 |
| IVR12_3 | IS30 | 2 | 1.54 | 2316 | 150023 |
| IVR12_4 | IS200/IS605 | 1 | 3.74 | 1583 | 42311 |
| IVR12_5 | IS200/IS605 | 1 | 4.69 | 570 | 12147 |
| PTA-126788 | total | 88 | 5.03 | 124827 | 2482713 |
