# Supplementary material for: In silico, in vitro and in vivo safety evaluation of Limosilactobacillus reuteri strains ATCC PTA-126787 & ATCC PTA-126788 for potential probiotic applications
Source: PLoS One. 2022 Jan 26;17(1):e0262663. doi: 10.1371/journal.pone.0262663 (PMC8791467; doi:10.1371/journal.pone.0262663)
Supplement: S5 Table — (DOCX) [file pone.0262663.s007.docx]

**S5 Table.** Predicted antimicrobial resistance genes in L. reuteri strains PTA-126787 and PTA-126788.

| **Strain** | **Chromosome** | **Location** | **Size (bp)** | **Identity (%)** | **Coverage (%)** | **Annotation** |
| --- | --- | --- | --- | --- | --- | --- |
| PTA-126787 | IU404_1 | 723,894 - 725,813 | 1920 | 100 | 99.96 | Tet(W) |
| PTA-126788 | IVR12_1 | 1,090,027 - 1,091,946 | 1920 | 100 | 99.96 | Tet(W) |
