# Supplementary material for: In silico, in vitro and in vivo safety evaluation of Limosilactobacillus reuteri strains ATCC PTA-126787 & ATCC PTA-126788 for potential probiotic applications
Source: PLoS One. 2022 Jan 26;17(1):e0262663. doi: 10.1371/journal.pone.0262663 (PMC8791467; doi:10.1371/journal.pone.0262663)
Supplement: S6 Table — (DOCX) [file pone.0262663.s008.docx]

**S6 Table.** Identified protein-coding genes putative for arginine deiminase pathway.

| **Locus tag** | **Gene function** | **Start** | **End** | **Strand** | **Size (bp)** |
| --- | --- | --- | --- | --- | --- |
| *Strain 3630* |  |  |  |  |  |
| IU404_01433 | Ornithine carbamoyltransferase (EC 2.1.3.3) | 1,356,883 | 1,357,890 | Forward | 1008 |
| IU404_01450 | Arginine deiminase (EC 3.5.3.6) | 1,372,477 | 1,373,709 | Forward | 1233 |
| IU404_01452 | Arginine/ornithine antiporter ArcD1 | 1,374,302 | 1,375,723 | Forward | 1422 |
| IU404_01453 | Arginine/ornithine antiporter ArcD1 | 1,375,781 | 1,377,178 | Forward | 1398 |
| IU404_01451 | Arginine repressor | 1,373,820 | 1,374,281 | Forward | 462 |
| IU404_02399 | Arginine repressor | 2,199,921 | 2,200,373 | Reverse | 453 |
| *Strain 3632* |  |  |  |  |  |
| IVR12_01894 | Ornithine carbamoyltransferase (EC 2.1.3.3) | 1,720,823 | 1,721,830 | Forward | 1008 |
| IVR12_01911 | Arginine deiminase (EC 3.5.3.6) | 1,736,417 | 1,737,649 | Forward | 1233 |
| IVR12_00724 | Arginine/ornithine antiporter ArcD1 | 608,272 | 609,693 | Forward | 1422 |
| IVR12_01913 | Arginine/ornithine antiporter ArcD1 | 1,738,242 | 1,739,663 | Forward | 1422 |
| IVR12_01914 | Arginine/ornithine antiporter ArcD1 | 1,739,721 | 1,741,118 | Forward | 1398 |
| IVR12_01912 | Arginine repressor | 1,737,760 | 1,738,221 | Forward | 462 |
