# Supplementary material for: In silico, in vitro and in vivo safety evaluation of Limosilactobacillus reuteri strains ATCC PTA-126787 & ATCC PTA-126788 for potential probiotic applications
Source: PLoS One. 2022 Jan 26;17(1):e0262663. doi: 10.1371/journal.pone.0262663 (PMC8791467; doi:10.1371/journal.pone.0262663)
Supplement: S7 Table — (DOCX) [file pone.0262663.s009.docx]

**S7 Table.** Putative genes in PTA-126787 and PTA-126788 involved in lactic acids production.

| **Gene function** | **Strain** | **Chromosome** | **Position** | **Locus Tag** | **Size (bp)** | **Strand** |
| --- | --- | --- | --- | --- | --- | --- |
| D-lactate dehydrogenase (EC 1.1.1.28) | PTA-126787 | IU404_1 | 531,116 - 532,129 | IU404_00583 | 1,014 | Reverse |
|  |  | IU404_1 | 606,464 - 607,549 | IU404_00662 | 1,086 | Forward |
|  |  | IU404_1 | 866,523 - 867,518 | IU404_00944 | 996 | Reverse |
|  |  | IU404_1 | 1,632,717 - 1,633,709 | IU404_01740 | 993 | Forward |
|  | PTA-126788 | IVR12_1 | 409,989 - 410,980 | IVR12_00498 | 992 | Reverse |
|  |  | IVR12_1 | 897,268 - 898,281 | IVR12_01036 | 1014 | Reverse |
|  |  | IVR12_1 | 972,605 - 973,690 | IVR12_01115 | 1086 | Forward |
|  |  | IVR12_1 | 1,230,501 - 1,231,496 | IVR12_01396 | 996 | Reverse |
| L-lactate dehydrogenase (EC 1.1.1.27) | PTA-126787 | IU404_1 | 72,380 - 73,318 | IU404_00084 | 939 | Forward |
|  |  | IU404_1 | 386,913 - 387,854 | IU404_00448 | 942 | Reverse |
|  |  | IU404_1 | 1,664,098 - 1,665,057 | IU404_01769 | 960 | Reverse |
|  |  | IU404_1 | 1,871,024 - 1,871,998 | IU404_02013 | 975 | Forward |
|  | PTA-126788 | IVR12_1 | 171,961 - 172,935 | IVR12_00207 | 975 | Reverse |
|  |  | IVR12_1 | 378,643 - 379,602 | IVR12_00467 | 960 | Forward |
|  |  | IVR12_1 | 752,015 - 752,956 | IVR12_00899 | 942 | Forward |
|  |  | IVR12_1 | 1,968,618 - 1,969,556 | IVR12_02186 | 939 | Reverse |

Note: IVR12_00498 encoding D-lactate dehydrogenase (EC 1.1.1.28) is a pseudogene due to frameshift.
