# Supplementary material for: In silico, in vitro and in vivo safety evaluation of Limosilactobacillus reuteri strains ATCC PTA-126787 & ATCC PTA-126788 for potential probiotic applications
Source: PLoS One. 2022 Jan 26;17(1):e0262663. doi: 10.1371/journal.pone.0262663 (PMC8791467; doi:10.1371/journal.pone.0262663)
Supplement: S8 Table — (DOCX) [file pone.0262663.s010.docx]

**S8 Table.** Identified protein-encoding genes putative for adhesion by L. reuteri strains PTA-126787 and PTA-126788.

| **Locus Tag** | **Size** | | **Gene Function** | **Strand** |
| --- | --- | --- | --- | --- |
| *Strain PTA-126787* | |  |  |  |
| IU404_01324 | | 178 | exonuclease | Reverse |
| IU404_02160 | | 82 | exopolysaccharide biosynthesis protein | Reverse |
| IU404_01408 | | 143 | F0F1 ATP synthase subunit epsilon | Forward |
| IU404_01134 | | 234 | class A sortase | Forward |
| *Strain PTA-126788* | |  |  |  |
| IVR12_01729 | | 178 | exonuclease | Reverse |
| IVR12_02549 | | 82 | exopolysaccharide biosynthesis protein | Forward |
| IVR12_01813 | | 143 | F0F1 ATP synthase subunit epsilon | Forward |
| IVR12_01541 | | 234 | class A sortase | Forward |
