# Supplementary material for: In silico, in vitro and in vivo safety evaluation of Limosilactobacillus reuteri strains ATCC PTA-126787 & ATCC PTA-126788 for potential probiotic applications
Source: PLoS One. 2022 Jan 26;17(1):e0262663. doi: 10.1371/journal.pone.0262663 (PMC8791467; doi:10.1371/journal.pone.0262663)
Supplement: S9 Table — (DOCX) [file pone.0262663.s011.docx]

**S9 Table.** Prophage regions in *L. reuteri* strains DSM 17938, PTA-126787 and PTA-126788.

| **Chromosome** | **Feature** | **Start** | **End** |
| --- | --- | --- | --- |
| *L. reuterii DSM 17938* |  |  |  |
| DSM_1 | prophage_region | 162588 | 187315 |
| DSM_1 | attL | 161835 | 161847 |
| DSM_1 | attR | 187473 | 187485 |
| DSM_1 | prophage_region | 472700 | 511947 |
| DSM_1 | attL | 472715 | 472755 |
| DSM_1 | attR | 513707 | 513747 |
| DSM_1 | prophage_region | 527443 | 568241 |
| DSM_1 | attL | 527458 | 527498 |
| DSM_1 | attR | 568450 | 568490 |
| DSM_1 | prophage_region | 876952 | 907794 |
| DSM_1 | attL | 876967 | 877007 |
| DSM_1 | attR | 917959 | 917999 |
| DSM_1 | prophage_region | 2093038 | 2137900 |
| DSM_1 | attL | 2093053 | 2093093 |
| DSM_1 | attR | 2134045 | 2134085 |
| *L. reuterii PTA-126788* |  |  |  |
| IU404_1 | prophage_region | 152457 | 167686 |
| IU404_1 | attL | 156168 | 156181 |
| IU404_1 | attR | 168138 | 168151 |
| IU404_1 | prophage_region | 290891 | 320979 |
| IU404_1 | attL | 289594 | 289607 |
| IU404_1 | attR | 319801 | 319814 |
| IU404_1 | prophage_region | 1720774 | 1762428 |
| IU404_1 | attL | 1718988 | 1719000 |
| IU404_1 | attR | 1763086 | 1763098 |
| IU404_1 | prophage_region | 1951294 | 1986058 |
| IU404_1 | attL | 1950720 | 1950733 |
| IU404_1 | attR | 1983717 | 1983730 |
| IU404_1 | prophage_region | 2129199 | 2187993 |
| IU404_1 | attL | 2132872 | 2132887 |
| IU404_1 | attR | 2184180 | 2184195 |
| IU404_1 | prophage_region | 2272336 | 2297075 |
| IU404_1 | attL | 2274787 | 2274924 |
| IU404_1 | attR | 2292319 | 2292456 |
| *L. reuterii PTA-126788* |  |  |  |
| IVR12_1 | prophage_region | 59142 | 102453 |
| IVR12_1 | attL | 60071 | 60088 |
| IVR12_1 | attR | 102593 | 102610 |
| IVR12_1 | prophage_region | 272211 | 335768 |
| IVR12_1 | attL | 270691 | 270703 |
| IVR12_1 | attR | 333993 | 334005 |
| IVR12_1 | prophage_region | 653929 | 686757 |
| IVR12_1 | attL | 654858 | 654875 |
| IVR12_1 | attR | 697380 | 697397 |
| IVR12_1 | prophage_region | 1870956 | 1891239 |
| IVR12_1 | attL | 1870254 | 1870339 |
| IVR12_1 | attR | 1906483 | 1906568 |
| IVR12_1 | prophage_region | 1953921 | 1961107 |
| IVR12_1 | attL | 1953219 | 1953304 |
| IVR12_1 | attR | 1989448 | 1989533 |
| IVR12_1 | prophage_region | 2044154 | 2082229 |
| IVR12_1 | attL | 2043452 | 2043537 |
| IVR12_1 | attR | 2079681 | 2079766 |
| IVR12_2 | prophage_region | 57619 | 72552 |
| IVR12_2 | attL | 56917 | 57002 |
| IVR12_2 | attR | 93146 | 93231 |
| IVR12_3 | prophage_region | 31417 | 68097 |
| IVR12_3 | attL | 30715 | 30800 |
| IVR12_3 | attR | 66944 | 67029 |
