# Supplementary material for: In silico, in vitro and in vivo safety evaluation of Limosilactobacillus reuteri strains ATCC PTA-126787 & ATCC PTA-126788 for potential probiotic applications
Source: PLoS One. 2022 Jan 26;17(1):e0262663. doi: 10.1371/journal.pone.0262663 (PMC8791467; doi:10.1371/journal.pone.0262663)
Supplement: S2 File — (PDF) [file pone.0262663.s013.pdf]

>*L. reuteri* PTA-126787 *tetW* sequence along with flanking gene sequences

TTAAGCTTCTTCGCCGCCAACCTTAACAACAGCTTTTGAAACCTTACCTAATTTACCACTAACAAAGTCTTCAACACCATCAAGGTCTAATTCGTGT
GAGAAGAGTGGTAGTGGGTCAACAACACCAGAAGAAAGAAGTGCAATTGAGTCTTCAAAAGTGTAAGGGTTAATGAAGGCCCCTTGAATTGTAAGTT
GTTTTTGGAATACATCGTAAGTGTTTACTGAGAACTTGTCATCAGGGTTACCAACACCAAACATTAATACTTGAGCACCACGAGCTGCAGCTGCAAG
TGCTTGTTCTTGAGTAGCTGGCAAACCAACCGCTTCAACAACGATGTCATATGCATCAGCAGGAATTTCTTCCTTAGTAGTGTTGATAGTCTTAACG
CCGAAGTGCTTCCGGTTGTTTTCCAACTTTTCATCTGAACGACCAGCTAAAGTAACTTCGTGAATACCACGAGCCTTCAAGATTTGTGCAAATAATT
GACCTTCAAAACCATCACCTAATACTAAAGCCTTTTGGTATGGGTGAGTTTCAAGTAAGTCAACACCGTGCATTGCACATGAAATTGGTTCAACAAC
GGCAGCTGCCTTTAATGAAACATCATCAGGAATTGGGTAAACAACTTTTGCAGGAGCAGTAAAGTATTCTTCAAAACCACCGTTACGGGTAACACCA
ACAGCATCAAGGTGTTCACATAATTCTGGGCGTTGAGTCCGACAATACTTACATTGACCACAGTAGATGTTAGGGTCAACGGTTACACGATCACCTG
GCTTAACGTTAGTAACTTCTGAACCAACTTTTGTAACTACCCCTGAGTTTTCATGACCTAAAACGATAGGAGGAACAGCTGATGCAGATCCCGGAAG
ACCGGCATAGAGCGCTTTATCAGTACCACAAATACCAGCGTAGGCAGTATGAATTAAGACTTCATCAGGCTTAATTTCAGGTTCTTTAATATCTTCA
ATTTCGAGTTGTTTTTACCTGTTAAAACAAGTGCCTTCATAATTAAAATACCTCTCTTGTGAATTTTATCATTTGTCAAGTGATAGTATAGTGTGA
AAACGCCTTAATTGTCAAACGTCCAACAATTATCTCACATTCTTTTCGCCATCATAAAACGTTAAACTCAGCAAATCCGAACCTAAATAAATCCCGG
TATAGAAGCATTTTCTAAAAAAGTGCAATTTTTAAATATTTTTTTCTTCACTAAGTTTATGCTATTTTTTTGTTATCACATAAATGGTTTTACTTTC
ATTTTTTAAATTACATAATTTCACAAGGCTTTTTATCCTATTTAGCGTTTTACATCAAGAAATAAAATGTTTCTACATATAAAGTAATAAATTCTAA
ATTTATATAGAAAATTTCTCCAACTTTCAGAAAGCGCTTAACTACCATTAGGCACGCCAAAACTTATCTCCAACCGCTCCTCAAAATCGCCCAAATG
GCCGTTATAACGGCTATTTTCCATTGCAGGATATATTTTTTATGTTACCCTAACAAAGTATTCGAACTTAGTTAATTATGATTTCCAATCTAATTA
ACAAAACTCAAACGAAAGGTGGATTTGGCGTGGACGACACAAAGAATCAACACCGGAAGCATAAACTTATTGAATATGCTAACGGTAAATCACTAGA
GGAAATCAACGGAACAGTTGAAGTTCCTCGTGGAAAAGGCTTCTGGCGAACATTATTCGCTTACTCTGGTCCCGGTGCATTAGTTGCTGTGGGTTAC
ATGGATCCAGGTAACTGGTCAACTTCAATTACTGGTGGACAGAGTTTCCAATATACCTTAATGACTACTATCTTGATTTCAAGTTTGATTGCGATGT
TGCTTCAATACATGGCGGCTAAACTCGGAATCGTGAGCCAAATGGACCTTGCTCAGGCAACACGGGCACGTACCGGTAAAGCATTAGGTATTATCTT
ATGGATTATGACTGAGTTGGCGATTATGGCTACTGATATCGCTGAAGTTATCGGGGCTGCTATTGCTTTAAACTTACTGTTCCACATTCCGTTGATC
CCATCTGTATTTATTACTGTTCTTGATGTTTTAGTACTGCTATTATTAACCAAGATCGGATTCCGGAAGATTGAAGCAATTGTTGCATGTTTGATCT
TGGTAATCTTGTTTGTTTTTGCTTACCAAGTTGCCTTATCTAACCCTAACTGGGGTGGCGTATTTATGGGTCTCCTTCCATCAGCTAAGGCAATTGC
CCAACATCCAGAAATTAGTGGTATTACTCCATTAACTGGATCATTAGGTATTATCGGTGCGACTGTTATGCCTCACAACCTATATCTCCACTCAGCA
ATTTCTCAAACTCGGAAGATTGACCACAATGATCTTGATAGCATTCGTCAAACTGTTCGTTTTACTACTTGGGATTCAAATATCCAATTATCCTTAG
CATTTATTGTTAACGCCCTTCTTTTAATCATGGGGGTTGCTGTTTTCAAGACTGGTGCTGTTCAAGATAGTTCTTTCTTCGGTTTATATGATGCCCT
GAATAACACTTCTATGCTTAGTAATCCAATCTTAATTGCGGTTGCTAAATCTGGTGTATTGTCAACTTTATTTGCAGTTGCTTTACTTGCCTCTGGT
CAAAATTCAACTATTACTGGAACATTAACCGGTCAAGTTATCATGGAAGGTTTCATTCATATGCGGATGCCTTTATGGGCACGACGGTTAGTTACCC
GGATTATTTCCGTTATTCCAGTTATTGCTTGTGTTGCTATGACGAGTGGTGAGAATACCATCCAACAACACACCGCCTTGAACCTTTTAATGGAAAA
TTCACAGGTATTCTTAGCTTTTGCCCTTCCATTCTCAATGTTGCCATTATTAATGATGACTAACAGTGAGGTTGAAATGGGTGAATTTAAGAATAGT
GGCTGGGTTAAGGCTTGCGGTTGGATCTCTGTAATTGCCCTTACCTTCTTAAATCTCTACAACCTTCCTGCAACTTATGAAGGTTTTGGTATTTGGG
CAAAAGGGACATCCGATGTTCTCGCATATATCACGATTATTGTCATTCTTGCTCTTCTTATCTGGACTTGTGTTGAACTCTACAAAGGGGATAAGCG
CTTTGCTGCTGAAGGTAAAGGATTCGGACAACGTGAAGCTCAAATGAAAGATTCAGCTGTAGAAGATTAAAATAAAAACCTGGAAGCAATGCTCATG
TACGGTAAAGAAGCAAGCAACCGAAAACAATAGATAGACCGCCAGCACTACACTATTCCGAACCAAAGACCAAAAGATAAGCATTTTGAGAAAATCT
AAACTTTTGGATTTTCCTACAATGCCGACTACGGCGGAATCCCTCCCACTCCTTATATATTTCTTTCTGTATACATTAAATTTGTATTTAGTAAAAT
GCAGACAACACCACGGATCGGCTTTTGGCTGGACAATTCCAACCAAACACCGCAGCAGACAGTAGAAACCATTCTGAACGTTAGGAAGCCGGTATGA
TTGTTACATATAAGGGGAAGAAAAATTTCTTTTAGATACTTGTTTTCCTAAAACTGATGTGATATAATAATTCAATTCCAGAAAAGGAGTAAAAAAT
ATGCGGCAAGTTATTCTTAAATAAAACTATAATCAAATAGTGGGAACAAAGGATTATGATAGTCCCTTTTGTAGGGGCTTAGTTTTTTGTACCCAAT
TTAAGAATACTTTTGCCTTATCAATTTTGACATATCCCCAAAAACAGCACTCACAAACAGGTGTATGCTGTATATGTGTATGTCCGCAAATTATCAT
CCCCAGTGGTAAAAGTATTTTACTGCTGGGGATTTTTATGCCCCTTCGGGGCAGTAAAGGGAGGACAATCACATGAAAATAATCAATATTGGAATTCT
TGCCCATGTAGACGCTGGAAAGACGACCTTGACGGAGAGCCTGCTATATGCCAGCGGAGCCATTTCAGAACCGGGGAGCGTCAAAAAAGGGACAACG
AGGACGGACACCATGTTTTTGGAGCGGCAGCGTGGGATTACCATTCAAGCGGCAGTCACTTCCTTCCAGTGGCACAGATGTAAAGTCAACATTGTGG
ATACGCCCGGCCACATGGATTTTTTGGCGGAGGTGTACCGCTCTTTGGCTGTTTTAGATGGGGCCATCTTGGTGATCTCCGCTAAAGATGGCGTGCA
GGCCCAGACCCGTATTCTGTTCCATGCCCTGCGGAAAATGAACATTCCCACCGTTATCTTTATCAACAAGATCGACCAGGCTGGCGTTGATTTGCAG
AGCGTGGTTCAGTCTGTTCGGGATAAGCTCTCCGCCGATATTATCATCAAGCAGACGGTGTCGCTGTCCCCGGAAATAGTCCTGGAGGAAAATACCG
ACATAGAAGCATGGGATGCGGTCATCGAAAATAACGATAAATTATTGGAAAAGTATATCGCAGGAGAACCAATCAGCCGGGAAAAACTTGTGCGGGA
GGAACAGCGGCGGGTTCAAGACGCCTCCCTGTTCCCGGTCTATTATGGCAGCGCCAAAAAGGGCCTTGGCATTCAACCGTTGATGGATGCGGTGACA
GGGCTGTTCCAACCGATTGGGGAACAGGGGAGCGCCGCCCTATGCGGCAGCGTTTTCAAGGTGGAGTATACAGATTGCGGCCAGCGGCGTGTCTATC
TACGGCTATACAGCGGAACGCTGCGCCTGCGGGATACGGTGGCCCTGGCCGGGAGAGAAAAGCTGAAAATCACAGAGATGCGTATTCCATCCAAAGG
GGAAATTGTTCGGACAGACACCGCTTATCCGGGTGAAATTGTTATCCTTCCCAGCGACAGCGTGAGGTTAAACGATGTATTAGGGGACCCAACCCGG
CTCCCTCGTAAAAGGTGGCGTGAGGACCCCCTCCCCATGCTGCGGACGTCGATTGCGCCGAAAACGGCAGCGCAAAGAGAACGGCTGCTGGACGCTC
TTACGCAACTTGCGGATACTGACCCGCTTTTGCGCTGCGAGGTGGATTCCATCACCCATGAGATCATTCTTTCTTTTTTGGGCCGGGTGCAGTTGGA
GGTTGTTTCCGCTTTGCTGTCGGAAAAATACAAGCTTGAAACAGTGGTAAAGGAACCCACCGTCATTTATAAGGAGCGGCCGCTCAAAGCAGCCAGC
CACACCATCCATATCGAGGTGCCGCCCAACCCGTTTTGGGCATCCATCGGACTGTCTGTTACACCACTCCCGCTTGGCTCCGGTGTACAATACGAGA
GCCGGGTTTCGCTGGGATACTTGAACCAGAGTTTTCAAAACGCTGTCAGGGATGGTATCCGTTACGGGCTGGAGCAGGGCTTGTTCGGCTGGAACGT
AACGGACTGTAAGATTTGCTTTGAATACGGGCTTTATTACAGTCCGGTCAGCACGCCGGCGGACTTCCGCTCATTGGCCCCGATTGTATTGGAACAG
GCATTGAAGGAATCAGGGACGCAACTGCTGGAACCTTATCTCTCCTTCACCCTCTATGCGCCCCGGGAATATCTTTCCAGGGCTTATCATGATGCAC
CGAAATACTGTGCCACCATCGAAACGGTCCAGGTAAAAAAGGATGAAGTTGTCTTTACTGGCGAGATTCCCGCCCGCTGTATACAGGCATACCGTAC
TGATCTGGCCTTTTACACCAACGGGCAGAGCGTATGCCTTACAGAACTGAAAGGGTATCAGGCCGCTGACGGCAAGCCAGTCATCCAGCCCCGCCGT
CCAAACAGCCGCCTGGACAAGGTGCGCTATATGTTTCAGAAGATAATGTAACTGCTTCCAGTTTTTTATTTTTGTAACGATTCTTTCACAAAATGTT
CATACTTGAACGGTATAGTATAGACAATCCCTAATATAAATAAACCCCTATTTTTCTTCTATACTATCCCCCTCAAAATAGCTATGAAGAAATACGA
AAAGTGGTCGGCCCATAATGAGTCGGCCATTTTTTAATACATCTAGGCTCACCATGGTATAATAAGGAACTAGATATATTACTCATTAAGTATGAGG
TGAAATCATTGAATAATAAAGATAATAATCAAAATAATAATAAGCGGCGACCTACTCGGGTTGAGCTTTATGACACTCCTAAGCCAGGAAAAGAAAA
GAAAAAACGTTCTTTCTTTAATCGTCAGCCTAAACAGACTACCCCTAACCATAAACATAAGACAGCATCCCCTAACAAGAAAAAGAATCCATATGGA
ATTTCACGTCGGATGTTCAAGGTCATTTTAGGCTGCCTATTAGTTATCTTAATTGCAACCATTGTTATTTTCTTAGTTAAACAACAGGACCCTGTCA
ATGTTGATAGCAATGACACTACTGTCTCCACAAGCACTTCATCACGCAAGAAGCATAAAAAGAGCAGCAGTAGCAGTCATAAGCATCGTTCAAGTTC
CAATGACAGTGATGACAATGATACTCAAGATACTTATAGTATTTCAACTCCTAGTCGTTCGGATAACACGCCTCAATCAAGTCATCCAACTAACCAA
CAACCAACTACTAACTCTTCTTCAACTCCACAACAAAACCAACCATCGCAGGAAGAACACCATGATCAAGGCAACAGCCAACAACAATCTACTTCAC
AACCAGCAAGCAGCAATTCTGGGTCACAGCCTAATCAAAGTTCGCAAAGTACTAGTTCCCAGTCAAACCCGCAACCAGTTCAACCTCATTCTCAAAA

TAATCAGCATTAATTTGGAATAAAGGGGTTGTCAATTAAAGTGGGCGTGCTATAATCAAGACAATCGATTGAAGGATATGCACCTTCAAAGTCCTCT
ACAGAGAGTAAGTGCCTTGAGTGGAAGCACTTGTGAGTGACAATGATGGTTGTACCACCTTCCCTTATTATTAGCCGAGATAAGGCTAGTCGGATCG
TGTCCGTTACCGCACCTTAGAGAGGACATTACTAGTGTGTCCTAAAGATGGGTGGAACCACGCTAATTTTTGTTAATTGACGTCCCTGATACTTTCA
ACATTGAAAGTATCAGGGACGTTTTTATTTATATTAAAGGAGAGATTTCTATGGCTCAGGTTGCTGTTATGTCCCCAGATGGATCAGTTAAGAAGAT
CGATCGGGATTCACAAGAAAGTTTAGAAGCATTACGTAAGCTTTCTGCATTAATGTTAAAGGCTGCATTAAAACAAGAATTTAAGGGTATTCGACTT
GGTGAAGCTGTCGCTGACGAAGATGGTTTCCACGTTGATTCTGATAAAGATAATCAACAAGTTTCTGCCGACGAATTACCAGCTCTTGAAGACGTAA
TCAAGGGAATGGCAAAGAACGATGTTAAGGTAGAATTTGTTGAAGTACCTGTAGAGGAAGCACTTGCCGAAGTAAAAGATGATCGTTTCTCAACTGA
ATTGATCAATGAAAATGCTAAAGACGGCAAAGTAGCAATGTACCAACTTGGTGATGTCAAAGCTGTTGCAGATGATGACATTCTTTTATATGGTAAC
GTTGTTAAAAACTTACGCCTTCTTTCTGTTGCTGGTGCTTACTGGAAAGGTATGTCTTCAAATCCAATGCTTCAACGGATTTACGGAACTGTCTTCT
ACAAGAAGGACGCATTAGAAGAAGACTTAAAGAAACGTCAAGAAGCTAAGGAACGTGACCACCGTGTTATCGGTAACCAACTCGACCTCTTCTTTGT
TGATCCTAAGGTTGGTGCCGGTTTACCATACTGGTTACCAAAAGGTGCTACTATTCGCCGGACAATCGAACGTTACATCATTGACCGGGAAGTTGCC
GATGGTTACCAACACGTTTATACTCCAGTCCTAATGAATCTTGATGCCTACAAGACTTCTGGTCACTGGGAACACTACCGCGACGATATGTTCCCAC
CAATGGACATGGGTGATGGCGAAATGCTTGAATTACGGCCAATGAACTGCCCAAGCCATATTCAAGTTTACAAGCACCATATTCGTTCATACCGTGA
TCTCCCATTACGAATTGCTGAACTTGGTATGATGCACCGTTATGAAAAATCAGGTGCTCTTTCTGGTCTTCAACGTGTTCGTGAAATGACTTTGAAC
GATGGCCACACTTTCGTTACCCTTGACCAAATTCGTTCTGAATTCGCAAAGATTTTGAAGTTGATCATGAGCGTTTACGAAGACTTTGATATTACTG
ACTACAGCTTCCGTCTTTCTCTTCGTGACCCTAAGAACGTTAAGAAGTACTATGCTAATGACGAAATGTGGGAAAAATCCCAATCAATGTTGAAATC
AGCAATGGACGATCTTAACCTTGATTATTATGAAGCTGAAGGTGAAGCTGCCTTCTATGGTCCAAAACTTGATATTCAAACTAAGACTGCTCTTGGG
AATGACGAAACAATGTCAACTATTCAACTTGACTTTATGCTTCCAGAACGATTCGGACTTTCCTACGTTGGTCAAGACGGTAAAGAACATCAACCAG
TTATGATTCACCGTGGTGTTGTTGGAACAATGGAACGGTTCATGGCTTACTTAACAGAAATTTACAAGGGTGCATTCCCAACTTGGTTAGCCCCAGA
ACAAGTCCACATTATCCCTGTTAACGAAGAAGCCCATGGTGAATACGCAGATAACCTCGCTAAGAAGATGAAGGCTGCTAACATTCGGGTTAATGTT
GACCACCGAAATGAAAAGATGGGCTACAAGATTCGTGAAGCTCAAACACAAAAGGTTCCATACACTCTTGTTGTTGGAGACGATGAAAAGAATAACA
ATGGTGTATCTGTTCGTAAGTACGGTGAAAAGGAACAAAATGAAATGAGTCAAGAAGCATTTATGAATGAAATTCTTGAAGATATTGCTTCTTACTC
CCGTGAAAAGTAA

tdh CDS
mntH CDS
tetW CDS
hypothetical protein CDS
thrS CDS

>*L. reuteri* PTA-126788 *tetW* sequence along with flanking gene sequences

TTAAGCTTCTTCGCCGCCAACCTTAACAACAGCTTTTGAAACCTTACCTAATTTACCACTAACAAAGTCTTCAACACCATCAAGGTCTAATTCGTGT
GAGAAGAGTGGTAGTGGGTCAACAACACCAGAAGAAAGAAGTGCAATTGAGTCTTCAAAAGTGTAAGGGTTAATGAAGGCCCCTTGAATTGTAAGTT
GTTTTTGGAATACATCGTAAGTGTTTACTGAGAACTTGTCATCAGGGTTACCAACACCAAACATTAATACTTGAGCACCACGAGCTGCAGCTGCAAG
TGCTTGTTCTTGAGTAGCTGGCAAACCAACCGCTTCAACAACGATGTCATATGCATCAGCAGGAATTTCTTCCTTAGTAGTGTTGATAGTCTTAACG
CCGAAGTGCTTCCGGTTGTTTTCCAACTTTTCATCTGAACGACCAGCTAAAGTAACTTCGTGAATACCACGAGCCTTCAAGATTTGTGCAAATAATT
GACCTTCAAAACCATCACCTAATACTAAAGCCTTTTGGTATGGGTGAGTTTCAAGTAAGTCAACACCGTGCATTGCACATGAAATTGGTTCAACAAC
GGCAGCTGCCTTTAATGAAACATCATCAGGAATTGGGTAAACAACTTTTGCAGGAGCAGTAAAGTATTCTTCAAAACCACCGTTACGGGTAACACCA
ACAGCATCAAGGTGTTCACATAATTCTGGGCGTTGAGTCCGACAATACTTACATTGACCACAGTAGATGTTAGGGTCAACGGTTACACGATCACCTG
GCTTAACGTTAGTAACTTCTGAACCAACTTTTGTAACTACCCCTGAGTTTTCATGACCTAAAACGATAGGAGGAACAGCTGATGCAGATCCCGGAAG
ACCGGCATAGAGCGCTTTATCAGTACCACAAATACCAGCGTAGGCAGTATGAATTAAGACTTCATCAGGCTTAATTTCAGGTTCTTTAATATCTTCA
ATTTCGAGTTGTTTTTTACCTGTTAAAACAAGTGCCTTCATAATTAAAATACCTCTCTTGTGAATTTTATCATTTGTCAAGTGATAGTATAGTGTGA
AAACGCCTTAATTGTCAAACGTCCAACAATTATCTCACATTCTTTTCGCCATCATAAAACGTTAAACTCAGCAAATCCGAACCTAAATAAATCCCGG
TATAGAAGCATTTTCTAAAAAAGTGCAATTTTTAAATATTTTTTTCTTCACTAAGTTTATGCTATTTTTTTGTTATCACATAAATGGTTTTACTTTC
ATTTTTTAAATTACATAATTTCACAAGGCTTTTTATCCTATTTAGCGTTTTACATCAAGAAATAAAATGTTTCTACATATAAAGTAATAAATTCTAA
ATTTATATAGAAAATTTCTCCAACTTTCAGAAAGCGCTTAACTACCATTAGGCACGCCAAAACTTATCTCCAACCGCTCCTCAAAATCGCCCAAATG
GCCGTTATAACGGCTATTTTCCATTGCAGGATATATTTTTTTATGTTACCCTAACAAAGTATTCGAACTTAGTTAATTATGATTTCCAATCTAATTA
ACAAAACTCAAACGAAAGGTGGATTTGGCGTGGACGACACAAAGAATCAACACCGGAAGCATAAACTTATTGAATATGCTAACGGTAAATCACTAGA
GGAAATCAACGGAACAGTTGAAGTTCCTCGTGGAAAAGGCTTCTGGCGAACATTATTCGCTTACTCTGGTCCCGGTGCATTAGTTGCTGTGGGTTAC
ATGGATCCAGGTAACTGGTCAACTTCAATTACTGGTGGACAGAGTTTCCAATATACCTTAATGACTACTATCTTGATTTCAAGTTTGATTGCGATGT
TGCTTCAATACATGGCGGCTAAACTCGGAATCGTGAGCCAAATGGACCTTGCTCAGGCAACACGGGCACGTACCGGTAAAGCATTAGGTATTATCTT
ATGGATTATGACTGAGTTGGCGATTATGGCTACTGATATCGCTGAAGTTATCGGGGCTGCTATTGCTTTAAACTTACTGTTCCACATTCCGTTGATC
CCATCTGTATTTATTACTGTTCTTGATGTTTTAGTACTGCTATTATTAACCAAGATCGGATTCCGGAAGATTGAAGCAATTGTTGCATGTTTGATCT
TGGTAATCTTGTTTGTTTTTGCTTACCAAGTTGCCTTATCTAACCCTAACTGGGGTGGCGTATTTATGGGTCTCCTTCCATCAGCTAAGGCAATTGC
CCAACATCCAGAAATTAGTGGTATTACTCCATTAACTGGATCATTAGGTATTATCGGTGCGACTGTTATGCCTCACAACCTATATCTCCACTCAGCA
ATTTCTCAAACTCGGAAGATTGACCACAATGATCTTGATAGCATTCGTCAAACTGTTCGTTTTACTACTTGGGATTCAAATATCCAATTATCCTTAG
CATTTATTGTTAACGCCCTTCTTTTAATCATGGGGGTTGCTGTTTTCAAGACTGGTGCTGTTCAAGATAGTTCTTTCTTCGGTTTATATGATGCCCT
GAATAACACTTCTATGCTTAGTAATCCAATCTTAATTGCGGTTGCTAAATCTGGTGTATTGTCAACTTTATTTGCAGTTGCTTTACTTGCCTCTGGT
CAAAATTCAACTATTACTGGAACATTAACCGGTCAAGTTATCATGGAAGGTTTCATTCATATGCGGATGCCTTTATGGGCACGACGGTTAGTTACCC
GGATTATTTCCGTTATTCCAGTTATTGCTTGTGTTGCTATGACGAGTGGTGAGAATACCATCCAACAACACACCGCCTTGAACCTTTTAATGGAAAA
TTCACAGGTATTCTTAGCTTTTGCCCTTCCATTCTCAATGTTGCCATTATTAATGATGACTAACAGTGAGGTTGAAATGGGTGAATTTAAGAATAGT
GGCTGGGTTAAGGCTTGCGGTTGGATCTCTGTAATTGCCCTTACCTTCTTAAATCTCTACAACCTTCCTGCAACTTATGAAGGTTTTGGTATTTGGG
CAAAAGGGACATCCGATGTTCTCGCATATATCACGATTATTGTCATTCTTGCTCTTCTTATCTGGACTTGTGTTGAACTCTACAAAGGGGATAAGCG
CTTTGCTGCTGAAGGTAAAGGATTCGGACAACGTGAAGCTCAAATGAAAGATTCAGCTGTAGAAGATTAAAATAAAAACCTGGAAGCAATGCTCATG
TACGGTAAAGAAGCAAGCAACCGAAAACAATAGATAGACCGCCAGCACTACACTATTCCGAACCAAAGACCAAAAGATAAGCATTTTGAGAAAATCT
AAACTTTTGGATTTTCCTACAATGCCGACTACGGCGGAATCCCTCCCACTCCTTATATATTTCTTTCTGTATACATTAAATTTGTATTTAGTAAAAT
GCAGACAACACCACGGATCGGCTTTTGGCTGGACAATTCCAACCAAACACCGCAGCAGACAGTAGAAACCATTCTGAACGTTAGGAAGCCGGTATGA
TTGTTACATATAAGGGGAAGAAAAATTTCTTTTAGATACTTGTTTTCCTAAAACTGATGTGATATAATAATTCAATTCCAGAAAAGGAGTAAAAAAT
ATGCGGCAAGTTATTCTTAAATAAAACTATAATCAAATAGTGGAACAAAGGATTATGATAGTCCCTTTTGTAGGGGCTTAGTTTTTTGTACCCAAT
TTAAGAATACTTTTGCCTTATCAATTTTGACATATCCCCAAAAACAGCACTCACAAACAGGTGTATGCTGTATATGTGTATGTCCGCAAATTATCAT
CCCCAGTGGTAAAAGTATTTTACTGCTGGGGATTTTTATGCCCTTCGGGGCAGTAAAGGGAGGACAATCACATGAAAATAATCAATATTGGAATTCT

TGCCCATGTAGACGCTGGAAAGACGACCTTGACGGAGAGCCTGCTATATGCCAGCGGAGCCATTTCAGAACCGGGGAGCGTCAAAAAAGGGACAACG
AGGACGGACACCATGTTTTTGGAGCGGCAGCGTGGGATTACCATTCAAGCGGCAGTCACTTCCTTCCAGTGGCACAGATGTAAAGTCAACATTGTGG
ATACGCCCGGCCACATGGATTTTTTGGCGGAGGTGTACCGCTCTTTGGCTGTTTTAGATGGGGCCATCTTGGTGATCTCCGCTAAAGATGGCGTGCA
GGCCCAGACCCGTATTCTGTTCCATGCCCTGCGGAAAATGAACATTCCCACCGTTATCTTTATCAACAAGATCGACCAGGCTGGCGTTGATTTGCAG
AGCGTGGTTCAGTCTGTTCGGGATAAGCTCTCCGCCGATATTATCATCAAGCAGACGGTGTCGCTGTCCCCGGAAATAGTCCTGGAGGAAAATACCG
ACATAGAAGCATGGGATGCGGTCATCGAAAATAACGATAAATTATTGGAAAAGTATATCGCAGGAGAACCAATCAGCCGGGAAAAACTTGTGCGGGA
GGAACAGCGGCGGGTTCAAGACGCCTCCCTGTTCCCGGTCTATTATGGCAGCGCCAAAAAGGGCCTTGGCATTCAACCGTTGATGGATGCGGTGACA
GGGCTGTTCCAACCGATTGGGGAACAGGGGAGCGCCGCCCTATGCGGCAGCGTTTTCAAGGTGGAGTATACAGATTGCGGCCAGCGGCGTGTCTATC
TACGGCTATACAGCGGAACGCTGCGCCTGCGGGATACGGTGGCCCTGGCCGGGAGAGAAAAGCTGAAAATCACAGAGATGCGTATTCCATCCAAAGG
GGAAATTGTTCGGACAGACACCGCTTATCCGGGTGAAATTGTTATCCTTCCCAGCGACAGCGTGAGGTTAAACGATGTATTAGGGGACCCAACCCGG
CTCCCTCGTAAAAGGTGGCGTGAGGACCCCCTCCCCATGCTGCGGACGTCGATTGCGCCGAAAACGGCAGCGCAAAGAGAACGGCTGCTGGACGCTC
TTACGCAACTTGCGGATACTGACCCGCTTTTGCGCTGCGAGGTGGATTCCATCACCCATGAGATCATTCTTTCTTTTTTGGGCCGGGTGCAGTTGGA
GGTTGTTTCCGCTTTGCTGTCGGAAAAATACAAGCTTGAAACAGTGGTAAAGGAACCCACCGTCATTTATAAGGAGCGGCCGCTCAAAGCAGCCAGC
CACACCATCCATATCGAGGTGCCGCCCAACCCGTTTTGGGCATCCATCGGACTGTCTGTTACACCACTCCCGCTTGGCTCCGGTGTACAATACGAGA
GCCGGGTTTCGCTGGGATACTTGAACCAGAGTTTTCAAAACGCTGTCAGGGATGGTATCCGTTACGGGCTGGAGCAGGGCTTGTTCGGCTGGAACGT
AACGGACTGTAAGATTTGCTTTGAATACGGGCTTTATTACAGTCCGGTCAGCACGCCGGCGGACTTCCGCTCATTGGCCCCGATTGTATTGGAACAG
GCATTGAAGGAATCAGGGACGCAACTGCTGGAACCTTATCTCTCCTTCACCCTCTATGCGCCCCGGGAATATCTTTCCAGGGCTTATCATGATGCAC
CGAAATACTGTGCCACCATCGAAACGGTCCAGGTAAAAAAGGATGAAGTTGTCTTTACTGGCGAGATTCCCGCCCGCTGTATACAGGCATACCGTAC
TGATCTGGCCTTTTACACCAACGGGCAGAGCGTATGCCTTACAGAACTGAAAGGGTATCAGGCCGCTGACGGCAAGCCAGTCATCCAGCCCCGCCGT
CCAAACAGCCGCCTGGACAAGGTGCGCTATATGTTTCAGAAGATAATGTAACTGCTTCCAGTTTTTTATTTTTGTAACGATTCTTTCACAAAATGTT
CATACTTGAACGGTATAGTATAGACAATCCCTAATATAAATAAACCCCTATTTTTCTTCTATACTATCCCCCTCAAAATAGCTATGAAGAAATACGA
AAAGTGGTCGGCCCATAATGAGTCGGCCATTTTTTAATACATCTAGGCTCACCATGGTATAATAAGGAACTAGATATATTACTCATTAAGTATGAGG
TGAAATCATTGAATAATAAAGATAATAATCAAAATAATAATAAGCGGCGACCTACTCGGGTTGAGCTTTATGACACTCCTAAGCCAGGAAAAGAAAA
GAAAAAACGTTCTTTCTTTAATCGTCAGCCTAAACAGACTACCCCTAACCATAAACATAAGACAGCATCCCCTAACAAGAAAAAGAATCCATATGGA
ATTTCACGTCGGATGTTCAAGGTCATTTTAGGCTGCCTATTAGTTATCTTAATTGCAACCATTGTTATTTTCTTAGTTAAACAACAGGACCCTGTCA
ATGTTGATAGCAATGACACTACTGTCTCCACAAGCACTTCATCACGCAAGAAGCATAAAAAGAGCAGCAGTAGCAGTCATAAGCATCGTTCAAGTTC
CAATGACAGTGATGACAATGATACTCAAGATACTTATAGTATTTCAACTCCTAGTCGTTCGGATAACACGCCTCAATCAAGTCATCCAACTAACCAA
CAACCAACTACTAACTCTTCTTCAACTCCACAACAAAACCAACCATCGCAGGAAGAACACCATGATCAAGGCAACAGCCAACAACAATCTACTTCAC
AACCAGCAAGCAGCAATTCTGGGTCACAGCCTAATCAAAGTTCGCAAAGTACTAGTTCCCAGTCAAACCCGCAACCAGTTCAACCTCATTCTCAAAA
TAATCAGCATTAATTTGGAATAAAGGGGTTGTCAATTAAAGTGGGCGTGCTATAATCAAGACAATCGATTGAAGGATATGCACCTTCAAAGTCCTCT
ACAGAGAGTAAGTGCCTTGAGTGGAAGCACTTGTGAGTGACAATGATGGTTGTACCACCTTCCCTTATTATTAGCCGAGATAAGGCTAGTCGGATCG
TGTCCGTTACCGCACCTTAGAGAGGACATTACTAGTGTGTCCTAAAGATGGGTGGAACCACGCTAATTTTTGTTAATTGACGTCCCTGATACTTTCA
ACATTGAAAGTATCAGGGACGTTTTTATTTATATTAAAGGAGAGATTTCTATGGCTCAGGTTGCTGTTATGTCCCCAGATGGATCAGTTAAGAAGAT
CGATCGGGATTCACAAGAAAGTTTAGAAGCATTACGTAAGCTTTCTGCATTAATGTTAAAGGCTGCATTAAAACAAGAATTTAAGGGTATTCGACTT
GGTGAAGCTGTCGCTGACGAAGATGGTTTCCACGTTGATTCTGATAAAGATAATCAACAAGTTTCTGCCGACGAATTACCAGCTCTTGAAGACGTAA
TCAAGGGAATGGCAAAGAACGATGTTAAGGTAGAATTTGTTGAAGTACCTGTAGAGGAAGCACTTGCCGAAGTAAAAGATGATCGTTTCTCAACTGA
ATTGATCAATGAAAATGCTAAAGACGGCAAAGTAGCAATGTACCAACTTGGTGATGTCAAAGCTGTTGCAGATGATGACATTCTTTTATATGGTAAC
GTTGTTAAAAACTTACGCCTTCTTTCTGTTGCTGGTGCTTACTGGAAAGGTATGTCTTCAAATCCAATGCTTCAACGGATTTACGGAACTGTCTTCT
ACAAGAAGGACGCATTAGAAGAAGACTTAAAGAAACGTCAAGAAGCTAAGGAACGTGACCACCGTGTTATCGGTAACCAACTCGACCTCTTCTTTGT
TGATCCTAAGGTTGGTGCCGGTTTACCATACTGGTTACCAAAAGGTGCTACTATTCGCCGGACAATCGAACGTTACATCATTGACCGGGAAGTTGCC
GATGGTTACCAACACGTTTATACTCCAGTCCTAATGAATCTTGATGCCTACAAGACTTCTGGTCACTGGGAACACTACCGCGACGATATGTTCCCAC
CAATGGACATGGGTGATGGCGAAATGCTTGAATTACGGCCAATGAACTGCCCAAGCCATATTCAAGTTTACAAGCACCATATTCGTTCATACCGTGA
TCTCCCATTACGAATTGCTGAACTTGGTATGATGCACCGTTATGAAAAATCAGGTGCTCTTTCTGGTCTTCAACGTGTTCGTGAAATGACTTTGAAC
GATGGCCACACTTTCGTTACCCTTGACCAAATTCGTTCTGAATTCGCAAAGATTTTGAAGTTGATCATGAGCGTTTACGAAGACTTTGATATTACTG
ACTACAGCTTCCGTCTTTCTCTTCGTGACCCTAAGAACGTTAAGAAGTACTATGCTAATGACGAAATGTGGGAAAAATCCCAATCAATGTTGAAATC
AGCAATGGACGATCTTAACCTTGATTATTATGAAGCTGAAGGTGAAGCTGCCTTCTATGGTCCAAAACTTGATATTCAAACTAAGACTGCTCTTGGG
AATGACGAAACAATGTCAACTATTCAACTTGACTTTATGCTTCCAGAACGATTCGGACTTTCCTACGTTGGTCAAGACGGTAAAGAACATCAACCAG
TTATGATTCACCGTGGTGTTGTTGGAACAATGGAACGGTTCATGGCTTACTTAACAGAAATTTACAAGGGTGCATTCCCAACTTGGTTAGCCCCAGA
ACAAGTCCACATTATCCCTGTTAACGAAGAAGCCCATGGTGAATACGCAGATAACCTCGCTAAGAAGATGAAGGCTGCTAACATTCGGGTTAATGTT
GACCACCGAAATGAAAAGATGGGCTACAAGATTCGTGAAGCTCAAACACAAAAGGTTCCATACACTCTTGTTGTTGGAGACGATGAAAAGAATAACA
ATGGTGTATCTGTTCGTAAGTACGGTGAAAAGGAACAAAATGAAATGAGTCAAGAAGCATTTATGAATGAAATTCTTGAAGATATTGCTTCTTACTC
CCGTGAAAAGTAA

tdh CDS
mntH CDS
tetW CDS
hypothetical protein CDS
thrS CDS

>Alignment of *tetW* of *L. reuteri* strains PTA-126787 and PTA-126788 along with other neighboring genes

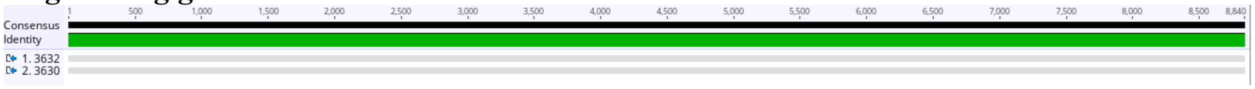

Note that *tetW* and the flanking regions for PTA-126787 and PTA-126788 are 100% identical.
